# Skin microbiome of coral reef fish is highly variable and driven by host phylogeny and diet

**DOI:** 10.1186/s40168-018-0530-4

**Published:** 2018-08-24

**Authors:** Marlène Chiarello, Jean-Christophe Auguet, Yvan Bettarel, Corinne Bouvier, Thomas Claverie, Nicholas A. J. Graham, Fabien Rieuvilleneuve, Elliot Sucré, Thierry Bouvier, Sébastien Villéger

**Affiliations:** 1 Marine Biodiversity, Exploitation and Conservation (MARBEC), Université de Montpellier, CNRS, IRD, IFREMER, Place Eugène Bataillon, Case 093, 34 095 Montpellier Cedex 5, France; 2 Laboratoire Ecologie Fonctionnelle et Environnement, Université de Toulouse, Toulouse, France; 3 Centre Universitaire de Formation et de Recherche de Mayotte, Dembéni, Mayotte, France; 4 Lancaster Environment Centre, Lancaster University, Lancaster, LA1 4YQ UK

**Keywords:** Tropical, Teleost, Microbiota, Phylogenetic diversity, Phylosymbiosis, Phylogenetic signal

## Abstract

**Background:**

The surface of marine animals is covered by abundant and diversified microbial communities*,* which have major roles for the health of their host*.* While such microbiomes have been deeply examined in marine invertebrates such as corals and sponges, the microbiomes living on marine vertebrates have received less attention. Specifically, the diversity of these microbiomes, their variability among species, and their drivers are still mostly unknown, especially among the fish species living on coral reefs that contribute to key ecosystem services while they are increasingly affected by human activities. Here, we investigated these knowledge gaps analyzing the skin microbiome of 138 fish individuals belonging to 44 coral reef fish species living in the same area.

**Results:**

Prokaryotic communities living on the skin of coral reef fishes are highly diverse, with on average more than 600 OTUs per fish, and differ from planktonic microbes. Skin microbiomes varied between fish individual and species, and interspecific differences were slightly coupled to the phylogenetic affiliation of the host and its ecological traits.

**Conclusions:**

These results highlight that coral reef biodiversity is greater than previously appreciated, since the high diversity of macro-organisms supports a highly diversified microbial community. This suggest that beyond the loss of coral reefs-associated macroscopic species, anthropic activities on coral reefs could also lead to a loss of still unexplored host-associated microbial diversity, which urgently needs to be assessed.

**Electronic supplementary material:**

The online version of this article (10.1186/s40168-018-0530-4) contains supplementary material, which is available to authorized users.

## Background

Lots of animals host abundant and diverse microbial communities, called microbiomes [1–5]. These microbiomes are crucial for their host’s fitness, as they regulate metabolism, enhance nutrients absorption, educate and regulate the immune system, and protect against pathogens [6]. Microbiomes are also distinct between host species [3, 7, 8], and these differences are sometimes related to host ecological traits; for instance, the gut microbiome of terrestrial vertebrates is linked to host diet [7]. Differences in microbiomes could also be correlated with evolutionary distance between hosts, with closely related species tending to host more similar microbiomes, a pattern called “phylosymbiosis” [9–11]. This pattern was reported not only for gut microbiomes of various animal clades, such as terrestrial mammals and insects [11, 12], but also for skin microbiomes of mammals belonging to *Artiodactyla* (even-toed ungulates including giraffe, goat, and camel) and *Perissodactyla* (odd-toed ungulates including horse and rhinoceros) [13]. Phylosymbiosis could be driven by an increased phenotypic divergence between hosts that are phylogenetically distinct [12], by vertical transmission of some microbial lineages across hosts generations [11], and/or coevolution of microbes with their host (e.g., a giant bacteria inhabiting surgeonfishes’ guts having phylogenetic relationships congruent with those of their hosts, i.e., cophylogeny) [14]).

By contrast to the numerous studies on gut microbiomes, the skin microbiomes of most animal taxa are underexplored, especially those of marine vertebrates which are surrounded by highly abundant and diverse planktonic microbes (viruses, bacteria, *Archaea*, and eukaryotes*)* in the seawater [15]. These planktonic reservoirs of microbes have potential to colonize vertebrate skin and potentially cause infections. Consequently, surface microbiomes of marine animals may be crucial for protection against pathogens. For instance coral surface mucus host bacterial species which are able to protect their host against pathogens by inhibiting enzymatic activities and secreting antimicrobial compounds [16–20].

However, the skin microbiome of marine fishes, which constitute the most diverse group of vertebrates [21], remains largely unknown with the exception of a few temperate species [3, 22]. More specifically, there is currently no knowledge about the factors explaining the diversity and the variability of skin microbiomes of tropical reef fishes. Many fish species are facing increasing threat, mainly due to human activities [23]. Understanding fish-microbes interactions in their natural environment is essential to further assess consequences of disturbances on such interactions, and consequences for host’s wild populations [24].

Here, we analyzed the prokaryotic microbiome of 44 fish species from the coral reefs of Mayotte Island (Western Indian Ocean) using metabarcoding of the V4 region of the 16S rRNA gene. We assessed the effect of host’s ecological traits and evolutionary legacy on the structure and diversity of its associated microbiome.

## Results

We sampled the skin microbiome of 138 individuals of 44 species of fish and 35 planktonic communities in a fringing reef and in an inner barrier reef around Mayotte Island (France). The two sampling sites were separated by 15 km. (See Additional file 1: S1 and the “Study area and sampling procedure” in the “Methods” section for more details). Fish species represented 5 orders and 22 families, including the main ecological groups dominating coral reefs. Biodiversity of microbial communities was assessed using phylogenetic entropy (Allen’s index), which takes into account both the phylogenetic affiliation of prokaryotic OTUs and their relative abundance [25]. Dissimilarity between microbial communities was assessed using W-Unifrac, which, as Allen’s index, is accounting for the relative abundance of phylogenetic lineages [26]. See “Computing phylogenetic diversity” in the “Methods” section for more details. As fish species were represented by one to six individuals (Additional file 1: S1), statistical tests assessing the effect of host species phylogenetic affiliation or ecological traits on fish skin microbiome were carried out using two different methodologies: Method A based on 999 random subsamples of 1 individual per fish species, and Method B based on averaged relative abundances of prokaryotic OTUs recovered on all individuals of each species (see “Determinants of dissimilarity between skin microbiomes” in the “Methods” section).

### Coral reef fishes host a high microbial diversity on their skin

**Fig. 1:**
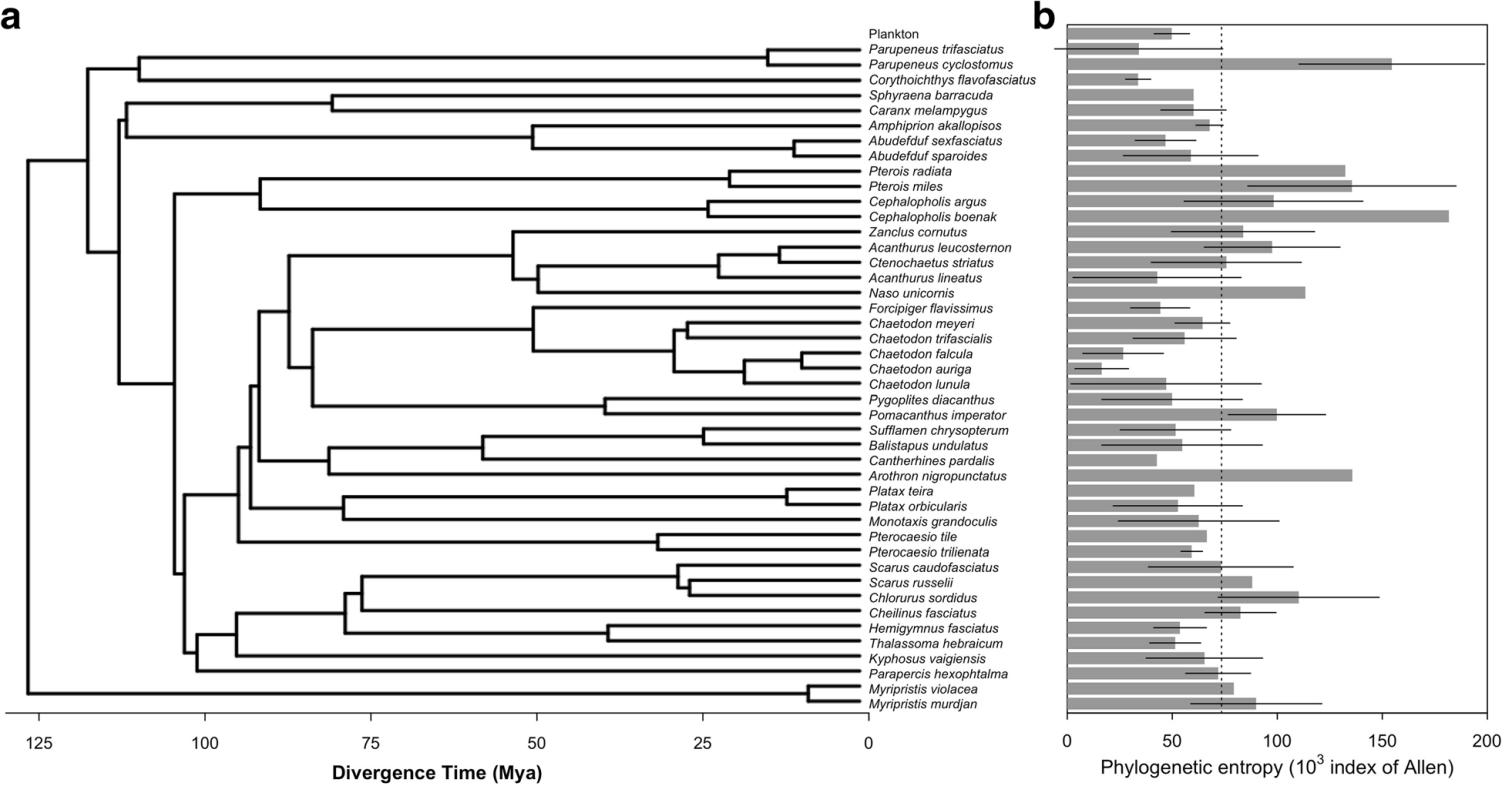
Phylogenetic tree and mean phylogenetic entropy of 44 fish species. **a** Phylogenetic tree relating all 44 fish species included in this study adapted from Rabosky et al. **b** Mean phylogenetic entropy of their skin-associated microbial community. Thick bars represent the mean of phylogenetic entropies across individuals belonging to the same fish species and horizontal segments represent the standard deviation across them. Dotted line indicates average phylogenetic entropy across all fish species. Phylogenetic entropy of planktonic communities is illustrated at the top of right panel

A total of 10,430 prokaryotic 97% similarity OTUs were found on fishes, representing 34 archaeal and bacterial classes and 19 phyla. In contrast, 2210 OTUs representing 17 classes and 11 microbial phyla were found in planktonic communities. Phylogenetic entropy of the skin microbiome of each fish individual was on average 1.4 times higher than in a planktonic sample (Kruskal-Wallis test, *P* = 0.003, Fig. 1 and Additional file 1: S2). The 35 planktonic communities combined hosted microbial phylogenetic entropy lower than all 100 randomly chosen of 35 fish microbiomes, which hosted on average 3 times higher phylogenetic entropy than planktonic communities (Additional file 1: S2).

**Fig. 2:**
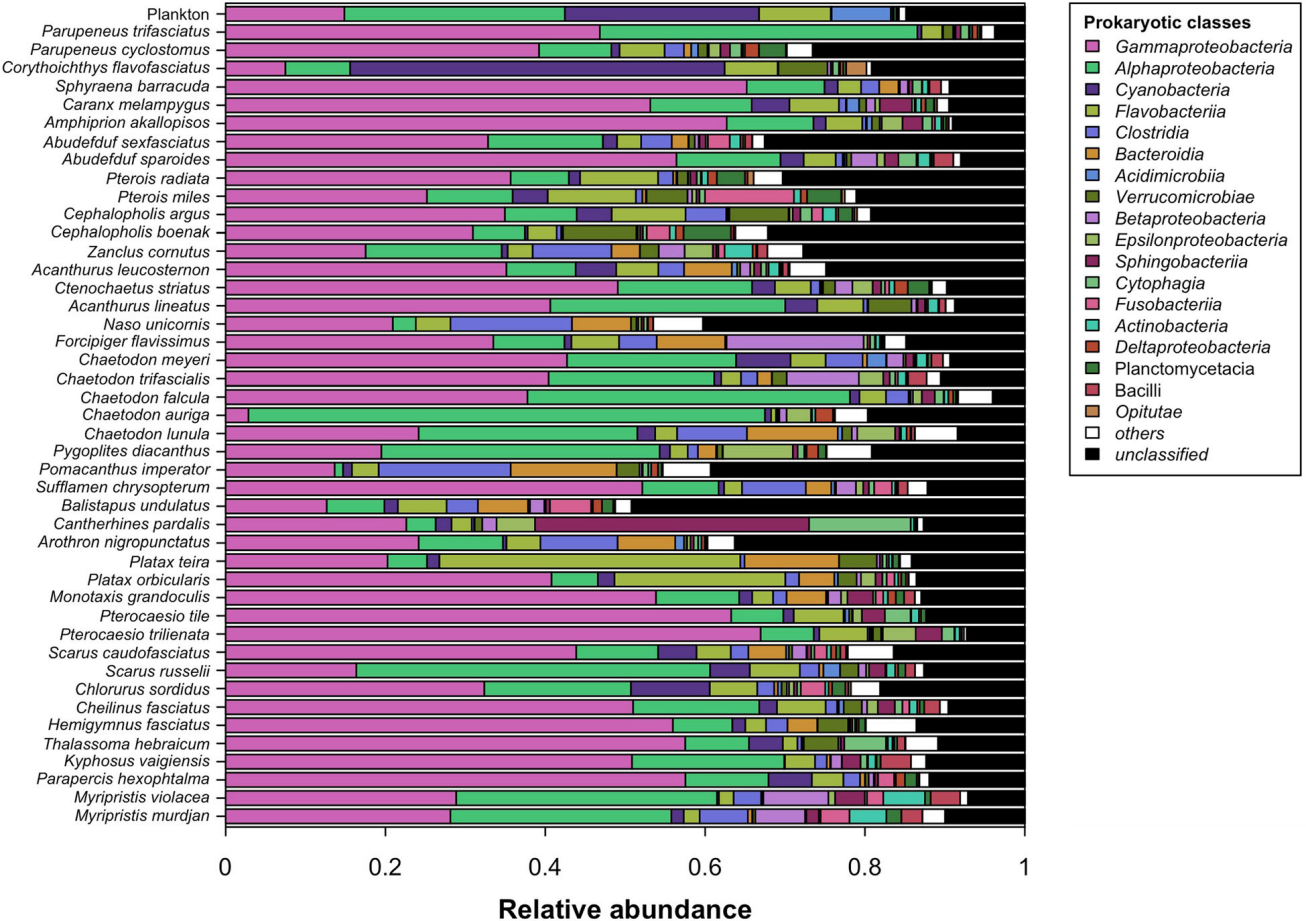
Mean class-level composition of fish skin microbiomes and planktonic communities. The 18 most abundant bacteria classes in all microbial communities are represented with colors. The mean composition of planktonic communities is indicated at the top. Taxonomic affiliation of OTUs was obtained from SILVA classification tool implemented in Mothur and refined using ARB parsimony tool and SILVA backbone tree. For classification without refinement, see Additional file 1: S12

**Fig. 3:**
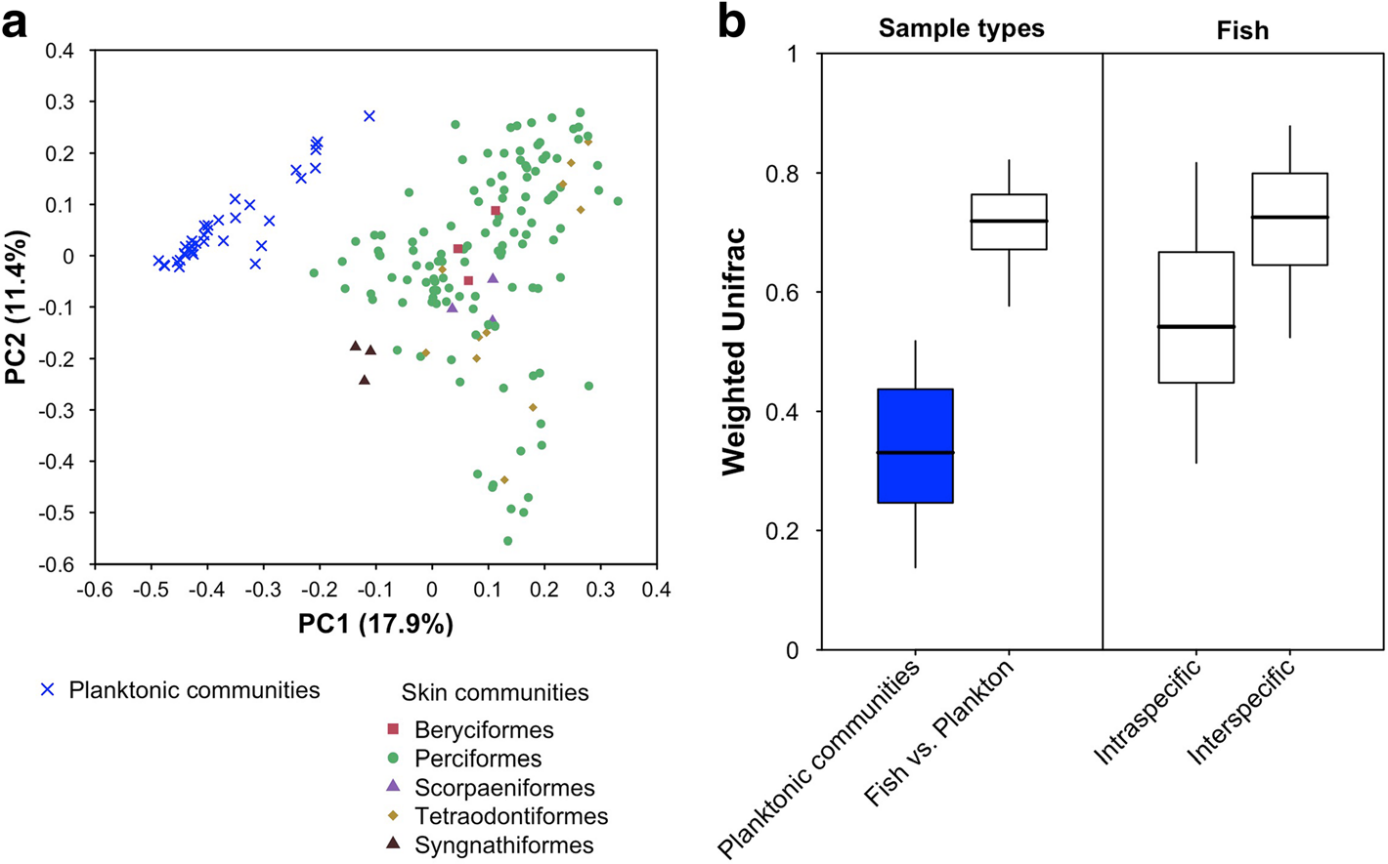
Dissimilarity between communities. **a** PCoA plot representing all fish skin microbiomes and planktonic communities included in this study, based on weighted phylogenetic dissimilarity values (W-Unifrac) between communities. Each dot represents one community (i.e., a water sample or a fish individual). Shape and color of dots indicate community type and fish taxonomic order. **b** W-Unifrac values**,** among planktonic communities (*n* = 35 samples), between fish skin microbiomes and planktonic communities (*n* = 173), between individuals of the same fish species (*n* = 34 species with more than 1 individual), and among individuals from different species (*n* = 44 species). Boxes represent the interquartile range dissimilarity values. Thick bars represent the median of dissimilarity values, and vertical segments extend to the fifth and the 95th percentiles of the distribution of values

In addition to these differences in phylogenetic diversity, fish skin microbiome has also significantly distinct phylogenetic structure than surrounding planktonic communities (PERMANOVA based on W-Unifrac, *P* = 0.001, and *R*^2^ = 0.14, Fig. 2 and Fig. 3). Fit of the neutral model from Sloan and co-workers [27] gave higher goodness of fit and migration rate on planktonic communities than on fish skin microbiomes (*R*^2^ = 0.62 and *m* = 0.58 for planktonic communities and *R*^2^ = 0.09 and *m* = 0.02 for fish skin microbiomes). Moreover, only 10% of OTUs found on fish skin were also detected in at least one planktonic community.

Fish skin microbiomes were significantly enriched in *Gammaproteobacteria* (14 ± 12% of abundance in plankton vs. 38 ± 24% on fishes), especially *Vibrionaceae* (1 ± 3% vs. 7 ± 11%) and *Altermonodales* (8 ± 10% vs. 10 ± 13%), *Rhizobiales* (0.01 ± 0.03% vs. 3 ± 5%), and *Clostridiales* (0.03 ± 0.04% vs. 3 ± 4%) compared to planktonic communities that were enriched in *Cyanobacteria (*24 ± 12% of abundance in water column vs. 4 ± 8% on fishes), *Rhodobacteraceae* (7 ± 4% vs. 6 ± 9%), and *Flavobacteriaceae* (9 ± 4% vs. 5 ± 7%) (Fig. 2 and Additional file 1: S3). Bacteria dominated both planktonic and skin-associated communities, as the 75 OTUs identified as Archaea cumulated 1.1% of abundance in planktonic communities and 0.8% in skin-associated communities. Thirty-seven percent of archaeal OTUs were affiliated to the phylum *Thaumarchaeota*, 17% to the phylum *Euryarchaeota*, and all other OTUs remained unclassified. *Thaumarchaeota* were mostly affiliated to the Marine Group I (9 OTUs out of 28 *Thaumarchaeota*) and South-African Gold Mine Group 1 (4 OTUs). *Euryarchaeota* were mostly classified into *Thermoplasmata* (5 OTUs out of 13 *Euryarchaeota*), *Methanomicrobia* (4 OTUs), and *Halobacteria* (3 OTUs). While not represented in Additional file 1: Figure S3 because of their small effect size, *Thaumarcheaota*, *Thermoplasmata*, and *Halobacteria* were significantly more abundant in fish skin microbiomes (see the Additional file 1: Table S3 for their respective effect sizes). Skin microbiomes of a few fish species (see Additional file 1: S1 and S7) were dominated by other prokaryotic classes (Fig. 2). For instance, *Chaetodon auriga* hosted the highest relative abundance in *Alphaproteobacteria* (64.6 ± 29% of relative abundance) and the lowest relative abundance in *Gammaproteobacteria* (2.8 ± 0.07%) in the entire dataset. *Corythoichthys flavofasciatus*, the only member of order *Syngnatiformes*, was the most enriched in *Cyanobacteria* (46.9 ± 13.1%) and second most depleted in *Gammaproteobacteria* (7.5 ± 0.03%). *Pomacanthus imperator* was the most enriched in *Clostridia* (16.5 ± 4.7%) and the most depleted in *Alphaproteobacteria* (1.0 ± 1.1%). *Naso unicornis* was the second most enriched in *Clostridia* (15.2%), and the most depleted in *Cyanobacteria* and *Alphaproteobacteria* (0% and 2.9%). The two *Epiphidae species* (*Platax teira* and *Platax orbicularis*) were the most enriched in *Flavobacteriia* (37.7% and 21.3 ± 29.3%). The only member of family *Monacanthidae* (*Cantherhines pardalis*) was the most enriched in *Sphingobacteriia* (34.3%).

Phylogenetic entropy varied significantly among fish species (Kruskal-Wallis test performed on Allen’s index of the 34 species represented by at least two individuals, *P* = 0.007). Phylogenetic entropy of fish skin microbiome varied among species of the same family from 1.02-fold factor (*Scorpaenidae*) to a 4.5-fold factor (*Mullidae*) and varied among individuals of the same species from 1.1-fold factor (*Pterocaesio trilienata*) to a 15.8-fold factor (*Chaetodon lunula*).

Using method A, interspecific differences of phylogenetic diversity of fish skin microbiome were significantly related to phylogenetic distances between fishes (*P* < 0.05) in 49% of the 999 subsamples (i.e., Moran’s *I* autocorrelation tests after subsampling of one fish individual per fish species; Moran’s *I =* 0.02 ± 0.0). Similar level of autocorrelation was obtained using method B (i.e., averaged microbiomes) and test was significant (*I* = 0.02, *P* = 0.05). None of the two methods raised a significant phylogenetic signal using Pagel’s Lambda (Additional file 1: S4).

### Dissimilarity among fish skin microbiomes

Dissimilarity among fish skin microbiomes was significantly higher than the one between planktonic communities (Kruskal-Wallis performed on W-Unifrac, *P* < 0.01, Fig. 3a), with pairwise W-Unifrac dissimilarities averaging 0.71 ± 0.11 for skin, and 0.34 ± 0.12 for plankton. No OTU was recovered on skin of all fish individuals. Interspecific W-Unifrac dissimilarities of skin microbiome were on average 1.3 times higher than intraspecific ones (Fig. 3b). Similarly, PERMANOVA performed on the 34 species represented by at least two individuals showed a significant effect of host species on its associated skin microbiome (*P* = 0.001, *R*^2^ = 0.44), demonstrating higher variability of skin microbiome between fish species compared to variability between individuals from the same species.

Additional PERMANOVAs performed on only fish species sampled on both reef types showed that host species had a higher effect size (*R*^2^ = 0.32) than reef type (*R*^2^ = 0.03, S5). Reef type (fringing vs. barrier) and environmental parameters (depth, swelling, weather, turbidity, temperature, conductivity, salinity, and total dissolved solids, see Additional file 1: S1) measured in both sites during sampling had a weak, although significant, effect on fish skin microbiome (separated PERMANOVAs performed on each parameter presented in S1, *P* < 0.05, *R*^2^ = 0.03 ± 0.00).

By contrast, planktonic communities showed higher dissimilarity between reef types (PERMANOVA performed on W-Unifrac, *P* = 0.001 *R*^2^ = 0.27) and stronger response to environmental parameters (effect sizes of separated PERMANOVAs, *P* < 0.05, *R*^2^ = 0.20 ± 0.09).

**Fig. 4:**
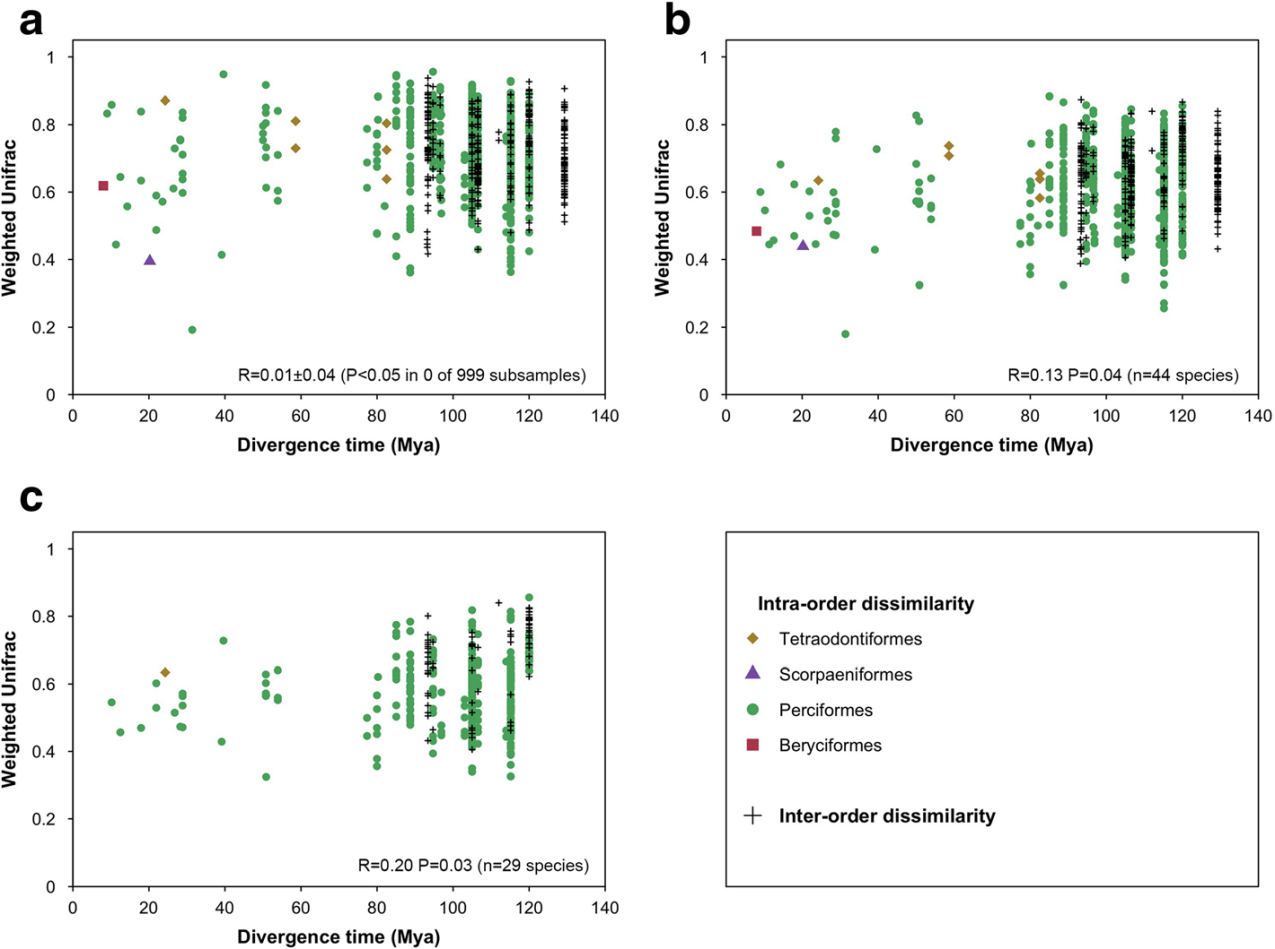
Phylogenetic dissimilarity (W-Unifrac) between skin-associated microbiomes of fishes against the divergence time between species. **a** Illustration of method A: one individual per fish species is represented. **b** Illustration of method B: W-Unifrac computed on averaged OTUs relative abundances across all individuals of each fish species. **c** Same as **b**, excepted that only species containing at least three individuals were represented. The result of the Mantel test corresponding to each methodology is displayed on each panel. Fishes are plotted as belonging to the same taxonomic order (dots) or belonging to different orders (‘+’ sign). Divergence time in millions of years ago (Mya). Note that intraspecific dissimilarities are not shown

Correlation between interspecific differences in the skin microbiomes and phylogenetic distances between host fish species raised different results depending on the methodology used (see “Determinants of dissimilarity between skin microbiomes” in the “Methods” section). Method A, involving subsamples of one fish individual per fish species before performing a Mantel test, did not detect any significant correlation between microbial and phylogenetic distances (Mantel test on W-Unifrac, *R* = 0.01 ± 0.04 and *P* < 0.05 in 0 of the 999 tested subsamples, Fig. 4a). Method B, which consisted in averaging microbial relative abundance across individuals of each fish species before computing W-Unifrac detected a significant correlation between microbial and phylogenetic distances (Mantel test: *R* = 0.13, *P* = 0.04 Fig. 4b). When considering only the 29 species containing at least three individuals correlation was even higher (*R* = 0.20, *P* = 0.03, Fig. 4c). However, both methods did not detect any correlation between microbial distances and host phylogeny at higher phylogenetic levels (Additional file 1: S6), even on the subset of 29 species containing at least 3 individuals.

Interspecific differences in the skin microbiomes (assessed using both methods A and B) were not significantly predicted by body size, schooling, period of activity, mobility, and position in the water column of the host. The only trait with a significant effect on fish skin microbiome was diet (PERMANOVAs performed on W-Unifrac; method A: *P* < 0.05 in 88% of 999 subsamples, *R*^2^ = 0.18; method B: *P* = 0.002 and *R*^2^ = 0.20) (Additional file 1: S7 and S8). However, the surface microbiome of sessile invertebrates sampled at the same period and on the same sites as fishes was not significantly closer to sessile invertebrates-eating fishes than to fishes having other diets (Additional file 1: S9).

### Assessing core skin microbiome of fish species

Among the 29 fish species that were sampled at least three times, from 0 to 110 core OTUs were recovered per species (i.e., OTUs that were recovered on the skin of all individuals of at least one species), making a total of 307 OTUs across all fish species (Additional file 1: S10). These OTUs were mainly *Gammaproteobacteria* (16% of core OTUs and 3.6 ± 5.7% of relative abundance when present), unclassified OTUs (14.7% of core OTUs and 0.08 ± 4.7% of relative abundance when present), and *Alphaproteobacteria* (13.7% of core OTUs and 1.9 ± 5.3% of relative abundance when present). Around 47% of fish core OTUs (making on average 29.1 ± 24% of cumulated relative abundance in fish skin microbiome) were also detected in planktonic communities, where they had a cumulated relative abundance of 80.6%.

The number of core OTUs and the number of individuals sampled were negatively correlated (Pearson’s correlation test, *P* = 0.005, rho = − 0.50). Additionally, there was no correlation between the average OTU richness on each species and the number of core OTUs recovered (Pearson’s correlation test, *P* = 0.26). Relative abundances of core OTUs recovered on each species were not correlated to host phylogeny (Moran’s *I* and Pagel’s Lambda on relative abundances of core OTUs, *P* = 1 across all core OTUs).

## Discussion

### Skin of coral reef fishes host a highly diverse microbiomes

Assessing the diversity of skin microbiome for 138 fishes inhabiting coral reefs revealed that a fish individual hosts as many as 600 OTUs and that the 44 fish species sampled host a total of 10,430 prokaryotic OTUs. Fish skin microbiomes hosted OTUs representing nearly twice more prokaryotic classes and phyla than planktonic communities. In addition to this high taxonomic diversity, the skin microbiome of each individual was phylogenetically more diverse than the planktonic community found in seawater (Fig. 1), and the phylogenetic entropy of the 35 combined planktonic communities sampled in our study hosted only the third of the phylogenetic entropy found on 35 randomly chosen fish individuals (Additional file 1: S2).

These results demonstrate that skin-associated microbiomes of tropical fishes host more microbial lineages than planktonic communities, and also that microbes abundant on skin are phylogenetically more distinct than those abundant in plankton. Such a high diversity of skin-associated microbial communities could be driven by the complexity of habitats available at fish surface, which are essentially alive tissues showing a specific and complex immune system [28], covered by a viscous, nutrient-rich mucus [29], whose composition is yet not well studied in numerous species. Tropical reefs are usually oligotrophic, and water column usually depleted in nutrients and organic matter. In these conditions, surface mucus may act as a growth media for microbes, as it has been hypothesized in the case of coral mucus [30]. In the case of fishes, estimates of cultural bacterial abundance were of 10^2^ to 10^4^ bacteria per square centimeter of skin [31], i.e., in approximately 0.003 to 0.01 mL of fish mucus [32], giving 10^4^ to 10^6^ culturable bacteria per milliliter of fish mucus, suggesting a possible enrichment of bacterial abundances compared to seawater (containing a total of 10^6^ bacteria per milliliter, whose 0.1 to 1% are culturable [15]). However, diversity of abiotic and biotic conditions on tropical fish skin still remain largely unknown and thus should be assessed in future studies to unravel niches available for microbes [22].

### Prokaryotic composition

Fish skin microbiome was largely dominated by Bacteria, totalizing more than 99% of OTU abundance, and especially *Gammaproteobacteria*. Previous studies also revealed high abundances of this bacterial class in teleost skin microbiome from temperate waters [3, 22], in surface mucus of corals [33], and on the skin of marine mammals [4, 34]. Besides reporting the dominant bacteria taxa present on fish skin, we also reported for the first time archaeal diversity of fish skin microbiome. The few archaeal lineages found on fishes included *Thaumarcheaota*, which is also the main archaeal phylum found on human skin [35]. Further investigations using specific primers are yet needed to explore this archaeal diversity more deeply, as primers used here are likely more efficient for recovery of bacterial diversity than archaeal diversity [36].

Fish skin microbiome was species-specific, both in terms of prokaryotic diversity (Fig. 2) and in terms of structure of the prokaryotic community (Fig. 3). To further test if species phylogenetic affiliation would drive both interspecific differences of microbial diversity and structure, we compared two different methods that seemed to be equally suitable to focus on drivers of interspecific variability of skin microbiome. The first one (method A), previously used by Groussin and co-workers [11], involved a random subsampling of one individual per species before statistical analyses. The second one (method B), previously used by Brooks and co-workers [12], involved averaging microbial relative abundances of prokaryotic OTUs found on individuals of the same species.

### Fish skin prokaryotic diversity

The two methods (A and B) yielded overall similar results concerning the drivers of interspecific differences of fish skin microbial diversity, identifying a slight trend of correlation between fish species phylogeny and prokaryotic phylogenetic entropy (i.e., phylogenetic signal, Fig. 2). However, Moran’s *I* autocorrelation measure was very low (Moran’s *I* = 0.02 for both methods), meaning that phylogenetic signal along fish phylogeny was weak. This weakness of phylogenetic signal was confirmed by measures using Pagel’s Lambda, which did not detect any significant phylogenetic signal (Additional file 1: S4).

The weakness of such correlation was partly driven by the high heterogeneity in microbial diversity between individuals belonging to the same family. For instance, microbiome phylogenetic entropy varied by a ~ 4-fold factor between the two *Mullidae* species *(Parupeneus trifasciatus* and *P. cyclostomus*, which diverged less than 15 Mya*)*. Therefore, fish species host different levels of microbial phylogenetic diversity, and these differences are only weakly phylogenetically conserved. This contrasts with a study on the whole microbiome of 20 marine sponges species, which showed a strong correlation between microbial diversity and host phylogeny [37]. To our knowledge, apart from ours, this study is the only one that tested such correlation. Differences of pattern may be related to the smaller phylogenetic scales studied here (8 to 130 Mya) compared to the divergence times between sponge species (up to 680 Mya in Easson and Thacker’s study according to http://timetree.org/). Moreover, the study from Easson and Thacker focused on the entire sponge microbiome, which is mainly located inside a tissue called *mesohyl* [38]. Such internal buffered microenvironment differs with fish surface, which is influenced by both surrounding biotic (e.g. grazing, viral lysis) and abiotic conditions (e.g. salinity), as well as plankton immigration [39].

### Fish skin prokaryotic structure

#### Phylosymbiosis

Besides diversity, phylogenetic structure of fish skin microbiome was also highly variable among fishes (Figs. 2 and 3). Strikingly, no OTU was recovered in all individuals. Such high variability of skin microbiome confirms findings reported for temperate fish species [3, 22]. Additionally, variability of skin microbiome was significantly lower between individuals from the same species than between individuals of different species, demonstrating a species-specificity of tropical fish skin microbiome (Fig. 3). Thus, similar to fishes from other ecosystems [3, 22], coral reef fish species host distinct microbial phylogenetic lineages. Previous studies reported phylosymbiosis for the gut microbiome of terrestrial animals (mammals [11], hominids [9], insects [12], birds [40]) and whole microbiomes of tropical sponges [37], and cophylogeny between surgeonfishes (*Acanthuridae*) and a bacterial symbiont [14]. To our knowledge, this is the first study investigating a possible phylosymbiosis pattern for the skin microbiome of marine fishes. The two statistical methods used identified contrasting results. The first one (method A) involving repeated random subsampling of one individual per species, revealed no significant phylosymbiosis pattern. The second one, involving averaging microbial relative abundances of prokaryotic OTUs found on individuals of the same species (method B), did detect a significant phylosymbiosis pattern (Fig. 4).

Fish skin microbiome is characterized by an important intraspecific microbial variability [22]. In our dataset, while being 1.3 times lower than the interspecific variability (Fig. 3), intraspecific variability might have blurred phylosymbiosis signal detection in method A, by considering one individual per species per subsample. However, the absence of any correlation between microbial distances and host phylogeny at higher phylogenetic levels using both methods (Additional file 1: S6), and the moderate *R*-value of Mantel test performed using method B (up to 0.2, which is lower than correlation coefficients found in gut microbiomes of terrestrial mammals by Groussin and coworkers using method A and similar statistical tests [11]), suggests that, if such a phylosymbiosis pattern exists in the skin microbiome of marine fishes, it remains low compared to other microbiomes.

Such weak phylosymbiosis pattern in fish skin microbiome may be related to the plasticity of fish immune system. Indeed, Malmstrom et al. [41] revealed that the number of copies of histo-incompatibility genes MHCI and MHCII, which encode proteins that detect non-self antigens and trigger an immune response, varies drastically among teleost fishes, and that differences between species were not strongly associated with their phylogenetic relationship. In addition, differences in skin immunology could occur between individuals (e.g., between starved and nourished individuals [42], between healthy and infected individuals [43], and between juveniles and adults [44]). Therefore, differences in the immune systems of fish could explain the high levels of both intra- and interspecific variability in skin microbiomes as well as the absence of a strong phylogenetic signal. However, it is now required to assess the phylogenetic conservatism of fish immune system, using, e.g., histo-compatibility genes sequencing and/or genomic approaches. The effect of immune system on fish skin microbiome also needs further investigation. A possibility would be the use of immunomic techniques (e.g., antibody microarrays) [45] combined with microbial 16S RNA sequencing in order to measure the effect of immune variations across individuals and species on active skin-associated microbes.

#### Environmental factors

Fishes included in this study were sampled less than 15 km apart, and reef type (fringing vs. barrier) explained less than 3% of the dissimilarity in skin microbiome of fishes found in both habitats, while fish species explained around 30% (Additional file 1: S5). Similarly, environmental parameters measured on both sites during the period of sampling explained around 3% of fish skin microbiome dissimilarity (Additional file 1: S1). By contrast, reef types explained around 20% of variability of planktonic communities (Additional file 1: S5). Fitting a neutral model for microbial dispersion on fish skin microbiomes and planktonic communities showed a much better fit of neutral model on planktonic communities than on skin microbiome (*R*^2^ = 0.62 and 0.09, respectively), and very high dispersion rate between water samples compared to the one between fish species (*m* = 0.58 and 0.02, respectively). Hence, contrary to planktonic communities, the variability in skin microbiome found among species is unlikely driven by the environmental factors measured and is thus rather driven by host-specific factors.

#### Ecological traits

We finally tested whether the phylogenetic structure of the skin microbiome could be predicted by key ecological traits of fishes (Additional file 1: S7). The only trait that yielded a consistently significant effect across both A and B methods was diet (*R*^2^ = 0.18 and 0.20 for methods A and B, respectively, Additional file 1: S8). Such an effect was not due to a transfer of microbial cells from sessile invertebrates to sessile invertebrates-eating fishes (Additional file 1: S9). Although it has been proven that diet shapes the gut microbiome of other vertebrates, including teleostean fishes, at both interspecific [7, 11, 46–48] and intraspecific scales [7, 49–51], this is the first report of an effect of species diet on the skin microbiome. An explanation could be an indirect transfer from fishes’ feces to their skin. However, the gut microbiome of the thousands of coral reef fishes [21, 52] is still largely unknown (but see [53] for *Acanthuridae* from the Red Sea). Another explanation would be that fishes having different diets produce different surface mucus. Accordingly, one study showed that different butterflyfishes produce distinct metabolites in their gill mucus, and that diet was the predominant factor explaining such differences [54]. Another study focusing on tropical reef fish also showed that gill microbiome was partially influenced by diet [55]. These findings suggest that the different metabolites present in fish alimentation sources may alter the mucus composition of the consumer, by modification of its physiology and/or by assimilation of certain metabolites and exudation in mucus, which would in turn alter microbial community composition in fish gills. Gill and skin mucus are both produced by goblet cells, share several similar components, and may thus be altered by similar pathways [56, 57]. Therefore, as in the case of gill mucus, diet may induce the production of distinct skin mucus, which may drive skin microbiome structure. Assessment of the metabolites present in skin mucus and the effect of fish diet at both inter- and intraspecific scales are now needed to confirm such hypothesis.

### Revealing the core microbiome of tropical reef fish species

Skin microbiome of marine fishes is a dynamic assemblage, which composition varies across time [58]. In that context, assessing the stable component of microbiomes, i.e., the core microbiome, is essential to characterize durable interactions between hosts fishes and their microbial partners, as well as predicting eventual alterations of a healthy community facing perturbations [59]. The core microbiome was defined as microbial OTUs that are present on all individuals of a given species [60] (Additional file 1: S10). We identified a total of 307 OTUs belonging to such core microbiomes, which belonged mainly to the *Gammaproteobacteria* class. The core fraction of fish microbiome contributed to on average 29.1 ± 24% of microbial abundance across the 29 species considered (Additional file 1: S10). We observed a strong negative correlation between the number of individuals sampled in each species and the number of core OTUs. Indeed, in fish species that were the most extensively sampled (5 individuals and more), only 0 to 10 core OTUs were recovered (while 2 to 110 core OTUs were recovered in other species), potentially indicating that a more extensive sampling of such fish species may prevent to recover the same OTU from all individuals, which is regularly observed in studies exploring core microbiome of other marine organisms [61] and highlights the high intraspecific variability of fish skin microbiome. Core microbiome is often considered to be adapted to niches at host’s surface that do not vary across host environmental range or condition, and/or that could be more likely vertically transmitted [61, 62], therefore, being more likely to follow a phylosymbiosis pattern. However, as here near 50% of core OTUs were also detected in planktonic communities, where they cumulated 80% of relative abundance (while only 10% of all OTUs detected in fishes were also detected in planktonic communities), such fraction of OTUs partly reflects the microbes able to colonize all environments available on a coral reef. Accordingly, we here detected no phylogenetic signal among any of these core OTUs, reinforcing the idea that they would more likely reflect the common environment of all fishes than a specific niche on fish skin.

## Conclusion

Here, we report that the high fish biodiversity on coral reefs supports a high biodiversity of microbial species because each fish species hosts a high and unique diversity of microbes. Comparing different methodologies, we also reveal that fish skin microbial diversity is driven by host phylogeny and diet. Contrasting results across methodologies giving a different weight to intraspecific variability of fish skin microbiome underline the importance of such variability that may prevent the detection of certain drivers if sampling effort is insufficient.

The weak phylosymbiosis pattern observed here has important consequences for the conservation of microbial diversity associated to fishes since protecting a few species of each clade does not prevent loss of a unique fraction of microbial diversity. These findings raise the need for a comprehensive assessment of the whole microbial biodiversity associated to coral reefs that are vanishing at an accelerating rate [63].

## Methods

### Study area and sampling procedure

Fish sampling was conducted on November 2015 (17th to 27th) on coral reefs around Mayotte Island (France), located in the western part of the Indian Ocean. The Mayotte Lagoon is the third largest lagoon in the world and houses 195 km of coral reefs and more than 700 fish species [64]. Fish were sampled from two sites in the South West of the lagoon: a fringing reef (S12°54′17.46″, E44°58′15.72″), and the inner slope of the barrier reef (S12°57′33.72″, E45°04′49.38″). Both sites are far from cities, were at a good ecological state at the time of sampling with more than 50% coral cover and abundant fish communities including predators such as groupers and barracudas. Environmental parameters were recorded on each site each sampling day (Additional file 1: S1).

The most abundant species of ecologically and phylogenetically contrasted fish families were sampled at each site (within a radius of 50 m), including representatives from the families *Acanthuridae*, *Balistidae*, *Chaetodontidae*, *Labridae*, *Pomacanthidae*, *Pomacentridae*, *Scaridae*, and *Scorpaenidae*. In order to take into account intraspecific variability of skin microbiome, up to five adult individuals of each species were sampled in each site.

In order to avoid contamination during sampling, fishes were caught using speargun and hook line and killed immediately after capture by cervical dislocation (following the European directive 2010/63/UE). Fishes were handled only by the mouth using a clamp and all participants wore gloves. After death, individuals were laid down, and skin microbiome was sampled by swabbing the entire untouched side of the body (from back of operculum to caudal peduncle, i.e., head not included) using buccal swabs (SK-2S swabs, Isohelix, UK). A total of 138 fishes were sampled for their skin microbiome. They belonged to 44 species with 29 species represented by at least three individuals (Additional file 1: S1) and 10 species represented by a single individual. Species belonged to 5 orders and 22 families, with 35 species belonging to Perciformes (Additional file 1: S1).

To assess planktonic diversity in the 2 sites, a total of 36 200-mL seawater samples were collected at the sea surface (9 samples) and at 30 cm from the seabed (9 samples), stored in an electric cooler, and filtrated at the end of the day through a 47 mm 0.2 μm polycarbonate membrane (Whatman, Clifton, USA). The membranes were then placed in sterile cryotubes. One surface water sample taken on the fringing reef could not be amplified during subsequent steps, and was removed, making a total of 35 water samples included in this study. All samples were stored at − 5 °C in an electric cooler during the day and remained frozen until DNA extraction.

### 16S rRNA gene amplification and sequencing

Swabs and water membranes were incubated during 30 min at 37 °C in 570 μL of lysis buffer from Maxwell® Buccal Swab LEV DNA kits (Promega Corporation, Madison, USA) and 2 μL of 37.5-KU.μL^− 1^ Ready-Lyse lysozyme™ (Epicenter Technologies, Madison, USA). Then, 30 μl of proteinase K (from manufacturer’s kit) were added and tubes were incubated overnight at 56 °C. The totality of the solution was then placed in the kit for extraction.

DNA extraction was performed using the Maxwell® 16 Bench-top extraction system following manufacturer’s instructions and eluted in 50 μL of elution buffer.

The V4 region of the 16S rRNA gene was amplified using the prokaryotic primers modified for Illumina sequencing 515F (5′-C TTT CCC TAC ACG ACG CTC TTC CGA TCT-GTG CCA GCM GCC GCG GTA A-3′) [65] and the modified version of 806R by Apprill et al. [66] (5′–G GAG TTC AGA CGT GTG CTC TTC CGA TCT-GGA CTA CNV GGG TWT CTA AT-3′), with PuRe *Taq* Ready-To-Go PCR Beads (Amersham Biosciences, Freiburg, Germany) using 1 μL of extracted DNA and 0.4 μM of each primer as follows: initial denaturation at 94 °C for 1 min followed by 35 cycles of 94 °C for 1 min, 55 °C for 1 min, and 72 °C for 1 min, ending with a final extension at 72 °C for 10 min. Equimolar amounts of sample DNA extracted from each sample site were separately pooled and sequenced in two separated runs by an external laboratory (INRA GeT-PlaGE platform, Toulouse, France) on an Illumina platform using the 2 × 250 bp MiSeq chemistry. Seven PCR blanks were included in each sequencing run in order to assess the presence of contaminants, which were removed during subsequent steps of sequence processing.

### Sequence processing to define OTUs and their phylogenetic relationships

Sequence processing was performed following the SOP of Kozich et al. for MiSeq [67], https://www.mothur.org/wiki/MiSeq_SOP, 2017) using Mothur [68]. After assembly of paired reads in each run, sequences of both runs were merged and sequences with an abnormal length (outside a range of 250–300 bp) were removed. Sequences were aligned along the SILVA reference database [69] release 128. Chimeras were removed using UCHIME [70]. Filtered sequences were then classified using the SILVA reference taxonomy and the non-prokaryotic ones were removed. 10,877 sequences from 173 samples were kept after the cleaning process, ranging from 2450 to 43,306 sequences per sample. After this, 2000 sequences were sub-sampled within each sample in order to correct the uneven sequencing efficiency among samples. Sequences were then grouped into operational taxonomic units (OTUs) using a 97% cut-off parameter, and the relative abundance of all OTUs was computed using number of sequences. Relative abundances of OTUs recovered from blank samples were then subtracted to their respective relative abundance in all other samples. Rarefaction curves obtained from all samples are provided in Additional file 1: S11. Non-parametric Chao’s coverage estimator was calculated using *entropart* R-package, and averaged 0.93 ± 0.05 across all samples.

The dominant sequence for each OTU was selected as reference and added into the SILVA reference phylogenetic tree (release 128) using the ARB parsimony insertion tool [71]. The full phylogenetic tree was then pruned using the *ape* R-package to remove all but the added sequences, while keeping the topology of the tree. A chronogram was then adjusted to the phylogenetic tree using PATHd8 [72]. The divergence time between *Archaea* and *Bacteria* was fixed at 3.8 Ga. The minimum divergence time between *Euryarchaeota* and other *Archaea* was set to 2.7 Ga [73], and the maximum age of apparition of *Thermoplamatales* was set to 2.32 Ga [73]. The minimum age of apparition of Cyanobacteria was set to 2.5 Ga [74]. The minimum divergence time between *Rickettsiales* and the rest of *Alphaproteobacteria* sequences was set at 1.6 Ga, following Groussin et al. [11]. Finally the divergence times between *Chromatiaceae* and other *Gammaproteobacteria* was set to minimum 1.64 Ga [75].

Fish skin microbiome and planktonic communities harbored high proportions of unclassified microbial taxa. Using the Mothur taxonomic affiliation method, as many as 60% of the 11,583 recovered OTUs in both fish skin microbiome and planktonic communities could not be classified at class level and 46% could not even be classified at phylum level. These OTUs ranged from 0 to 34% of total abundance in a sample. We refined the taxonomic affiliation of the most frequent unclassified OTUs (i.e., the 571 OTUs that were unclassified at phylum level and that were recovered in at least 5 samples and/or contributed to more than 1% of abundance in at least one sample) using the ARB parsimony insertion tool and the SILVA backbone tree (v128) (Fig. 3). One hundred seventy seven of them belonged to classes that were not detected during OTU classification by Mothur. See Additional file 1: S12 for the prokaryotic classes’ relative abundances using Mothur’s classification and the refined classification of the 571 initially unclassified OTUs.

### Computing phylogenetic diversity

We measured phylogenetic entropy accounting for the relative abundance of OTUs, using Allen’s index [25], which is a phylogenetic extension of Shannon’s taxonomic entropy index. Allen’s index was computed using our own R-function (https://github.com/marlenec/chao, *q* = 1) based on the entropart R-package [76]. Allen’s index increases when the most abundant OTUs are phylogenetically distant.

Phylogenetic dissimilarities between pairs of microbial assemblages were assessed using the abundance weighted Unifrac index (W-Unifrac) computed using the GUniFrac R-package [77]. Phylogenetic dissimilarity indices accounting for structure ranges from 0 when assemblages share the same dominant phylogenetic lineages to 1 when assemblages are or dominated by phylogenetically distant OTUs.

### Fish phylogeny and ecological traits

Phylogenetic relationships between studied fish species were extracted from a published time-calibrated phylogeny containing 7822 fish species, covering all Actinopterygian orders [78]. Out of the 44 fish species, 13 were not present in the phylogenetic tree and were manually grafted next to their closest species accordingly to literature. One species (*Cephalopholis argus*) was incorrectly branched next to *Scaridae* in the initial tree and was therefore also grafted next to its closest relative (see Additional file 1: S13).

The ecology of the 44 species was described using a set of 6 categorical traits describing body size at fish maturity, mobility, period of activity, schooling behavior, position in water column, and diet. Values were taken from a global database of functional traits for 6316 tropical reef fishes [79]. The distribution of trait values among the 44 studied species is described in Additional file 1: S7.

### A and B methodologies used to test interspecific drivers of fish skin microbiomes

Measures of the correlation between fish ecological traits or phylogeny and the diversity and dissimilarity of their associated microbiomes were performed using two complementary methodologies. Method A involved computing diversity indices and statistical tests on 999 random subsamples of 1 individual for each of the 44 species to account for intraspecific variability. Method B involved averaging prokaryotic OTUs relative abundance observed among individuals from each species before computing diversity and dissimilarity indices and associated statistical tests on these species microbiomes.

### Determinants of microbial diversity

The comparison of phylogenetic entropy obtained in planktonic samples and fish skin microbiomes was done using a Kruskal-Wallis test (999 permutations) in *vegan* R-package. To fairly compare planktonic diversity to the one of the fish skin microbiome, we computed the phylogenetic entropy of 35 randomly chosen individuals (100 bootstrap replicates) before comparison to one found in the whole planktonic community (35 samples) (see Additional file 1: S2).

The comparison of phylogenetic richness and phylogenetic entropy between fish species was done using a Kruskal-Wallis test (999 permutations) based on the 34 fish species that contained at least 2 individuals (128 individuals).

To test if closely related fish species have more similar levels of phylogenetic entropy values than expected by chance, we computed both Moran’s *I*, which is used as an autocorrelation measure of trait variation along a phylogenetic tree, and Pagel’s Lambda, which is a measure of conformity of observed traits distribution to a model of Brownian trait evolution. To calculate Moran’s *I*, we used the inverse of divergence times between fish species as a measure of phylogenetic proximity [80]. Then, Moran’s *I* observed value was compared to the ones obtained when shuffling diversity values 999 times on the phylogenetic tree using *adephylo* R-package. Observed Pagel’s Lambda was calculated using the function ‘fitContinuous’ from *geiger* R-package and compared to the ones obtained when shuffling diversity values 500 times (due to extensive calculation time) on the phylogenetic tree. These tests were performed using the two methodologies described above (see the “A and B methodologies used to test interspecific drivers of skin microbiomes” section).

### Determinants of dissimilarity between skin microbiomes

The comparison of the structure of fish-associated microbial communities and the planktonic ones was performed on the full dataset using a permutational multivariate ANOVA (PERMANOVA) performed on dissimilarity values (W-Unifrac) using *vegan* R-package. To assess how each microbial clade contributed to the dissimilarity between planktonic and skin-associated microbial communities, we performed a LefSe analysis [81]. LefSe provides linear discriminant analysis (LDA) scores for the bacterial clades contributing the most to the differences between communities (Additional file 1: S3).

The assessment of the effect of fish species on skin microbial community structure was done using a PERMANOVA on the dissimilarities between individuals (*n* = 128) of the 34 species that contained at least 2 individuals. To assess the effect of reef type on the fish skin microbiome, we performed a PERMANOVA on the 16 species for which we sampled at least 1 representative on both reef types (total of 74 individuals). To compare the effects of reef type and fish species on its microbiome, both factors, as well as the interaction between them, were included in the analysis (Additional file 1: S5). The effect of environmental parameters measured on the field the day of sampling of each individual (minimum and maximum depth, height of the swells, sunshine, water turbidity, ambient and water temperatures, and water’s conductivity, salinity, and total dissolved solids, Additional file 1: S1) was measured using a separated PERMANOVA for each parameter.

In order to test whether fish skin microbial composition could be explained by a neutral model of species dispersion and extinction, we fitted the neutral model from Sloan et al. [27] on OTU abundances found in skin fish microbiome and planktonic communities using the R-script from Burns and coworkers [82]. This analysis was performed using method B only.

The correlation between interspecific dissimilarities and hosts’ phylogeny (phylosymbiosis) was measured using Mantel tests based on Pearson’s coefficient, using *vegan* R-package and 999 permutations. This analysis was performed using the two methods described above (A and B).

In order to assess the effects of host phylogeny at higher phylogenetic levels than OTUs, we used the beta diversity through time (BTTD) approach developed by Groussin et al. [11], which computes various beta-diversity indices at different time periods (slices) along the bacterial phylogenetic tree. We went back in time this way until 900 Mya, which corresponds approximately to divergence between bacterial orders, and computed Bray-Curtis index at each slice of 100 Mya. At each slice, correlation between pairwise beta-diversity values and host phylogeny was tested using a Mantel test based on Pearson’s coefficient and 999 permutations. This analysis was performed using both methods described above (see the “A and B methodologies used to test interspecific drivers of skin microbiomes” section). For this analysis, due to extensive computation time, method A was performed using only 500 subsamples instead of 999.

The effect of fish ecological traits was assessed using PERMANOVAs, using both methods described above (see the “A and B methodologies used to test interspecific drivers of skin microbiomes” section). The ecological traits were ordered in the model according to their independent contribution (greatest to least) to the total variability.

For all analyses involving dataset subsampling (method A), results were reported as the percentage of significant *P* values (*P* < 0.05) obtained in all subsamples, and when useful, the mean standard deviation of the statistic among all subsamples.

### Core microbiomes

Core OTUs for each species were defined as OTUs that were shared by all individuals of the same species (Additional file 1: S10). Correlation between the number of core OTUs and the number of individuals sampled and the average OTUs richness of each species was measured using two separate Pearson’s correlation tests.

To test if closely related fish species had more similar levels core OTUs abundances than expected by chance, we computed both Moran’s *I* and Pagel’s Lambda on each core OTUs relative abundance distribution and compared observed values to the ones obtained when shuffling relative abundances on fish phylogenetic tree (*n* = 999 and 500 permutations for Moran’s *I* and Pagel’s Lambda, respectively), see the “Determinants of microbial diversity” section for more details. *P* values were subsequently corrected for multiple testing using Bonferroni formula.

## Additional files

Additional file 1: Supplementary information S1 to S13. (DOCX 8832 kb)

Additional file 2: Supplementary material containing OTU table, list of corresponding reference sequence for each OTU, and sample metadata. (ZIP 946 kb)
