# Supplementary material for: Skin microbiome of coral reef fish is highly variable and driven by host phylogeny and diet
Source: Microbiome. 2018 Aug 24;6:147. doi: 10.1186/s40168-018-0530-4 (PMC6109317; doi:10.1186/s40168-018-0530-4)

**Skin microbiome of coral reef fish is highly variable and driven by host phylogeny and diet**

Marlène Chiarello*^1,2^, Jean-Christophe Auguet^1^, Yvan Bettarel^1^, Corinne Bouvier^1^, Thomas Claverie^1,3^, Nicholas AJ Graham^4^, Fabien Rieuvilleneuve^1^, Elliot Sucré^1,3^, Thierry Bouvier^1#^, and Sébastien Villéger^1#^

^#^ co-senior authorship

*Corresponding author: [marlene.chiarello@gmail.com](mailto:marlene.chiarello@gmail.com)

**Affiliations:**

^1^Marine Biodiversity, Exploitation and Conservation (MARBEC), Université de Montpellier, CNRS, IRD, IFREMER, Place Eugène Bataillon, Case 093, 34 095 Montpellier Cedex 5, France

^2^Université de Toulouse, Laboratoire Ecologie Fonctionnelle et Environnement, Toulouse, France

^3^Centre Universitaire de Formation et de Recherche de Mayotte, Dembéni, Mayotte, France

^4^Lancaster Environment Centre, Lancaster University, Lancaster, LA1 4YQ, UK

**Supplementary Information S1: species sampling and environmental parameters**

| Order | Family | Species | Number of individuals |
| --- | --- | --- | --- |
| Beryciformes | *Holocentridae* | *Myripristis murdjan* | 2 |
| Beryciformes | *Holocentridae* | *Myripristis violacea* | 1 |
| Perciformes | *Acanthuridae* | *Acanthurus leucosternon* | 5 |
| Perciformes | *Acanthuridae* | *Acanthurus lineatus* | 3 |
| Perciformes | *Acanthuridae* | *Ctenochaetus striatus* | 7 |
| Perciformes | *Acanthuridae* | *Naso unicornis* | 1 |
| Perciformes | *Caesionidae* | *Pterocaesio tile* | 1 |
| Perciformes | *Caesionidae* | *Pterocaesio trilienata* | 2 |
| Perciformes | *Carangidae* | *Caranx melampygus* | 3 |
| Perciformes | *Chaetodontidae* | *Chaetodon auriga* | 2 |
| Perciformes | *Chaetodontidae* | *Chaetodon falcula* | 6 |
| Perciformes | *Chaetodontidae* | *Chaetodon lunula* | 4 |
| Perciformes | *Chaetodontidae* | *Chaetodon meyeri* | 3 |
| Perciformes | *Chaetodontidae* | *Chaetodon trifascialis* | 3 |
| Perciformes | *Chaetodontidae* | *Forcipiger flavissimus* | 3 |
| Perciformes | *Ephippidae* | *Platax orbicularis* | 5 |
| Perciformes | *Ephippidae* | *Platax teira* | 1 |
| Perciformes | *Kyphosidae* | *Kyphosus vaigiensis* | 3 |
| Perciformes | *Labridae* | *Cheilinus fasciatus* | 3 |
| Perciformes | *Labridae* | *Hemigymnus fasciatus* | 3 |
| Perciformes | *Labridae* | *Thalassoma hebraicum* | 3 |
| Perciformes | *Lethrinidae* | *Monotaxis grandoculis* | 5 |
| Perciformes | *Mullidae* | *Parupeneus cyclostomus* | 2 |
| Perciformes | *Mullidae* | *Parupeneus trifasciatus* | 3 |
| Perciformes | *Pinguipedidae* | *Parapercis hexophtalma* | 3 |
| Perciformes | *Pomacanthidae* | *Pomacanthus imperator* | 3 |
| Perciformes | *Pomacanthidae* | *Pygoplites diacanthus* | 6 |
| Perciformes | *Pomacentridae* | *Abudefduf sexfasciatus* | 3 |
| Perciformes | *Pomacentridae* | *Abudefduf sparoides* | 4 |
| Perciformes | *Pomacentridae* | *Amphiprion akallopisos* | 3 |
| Perciformes | *Scaridae* | *Chlorurus sordidus* | 6 |
| Perciformes | *Scaridae* | *Scarus caudofasciatus* | 4 |
| Perciformes | *Scaridae* | *Scarus russelii* | 1 |
| Perciformes | *Serranidae* | *Cephalopholis argus* | 6 |
| Perciformes | *Serranidae* | *Cephalopholis boenak* | 1 |
| Perciformes | *Sphyraenidae* | *Sphyraena barracuda* | 1 |
| Perciformes | *Zanclidae* | *Zanclus cornutus* | 6 |
| Scorpaeniformes | *Scorpaenidae* | *Pterois miles* | 2 |
| Scorpaeniformes | *Scorpaenidae* | *Pterois radiata* | 1 |
| Syngnathiformes | *Syngnathidae* | *Corythoichthys flavofasciatus* | 3 |
| Tetraodontiformes | *Balistidae* | *Balistapus undulatus* | 3 |
| Tetraodontiformes | *Balistidae* | *Sufflamen chrysopterum* | 6 |
| Tetraodontiformes | *Diodontidae* | *Arothron nigropunctatus* | 1 |
| Tetraodontiformes | *Monacanthidae* | *Cantherhines pardalis* | 1 |
|  |  | **Total Individuals** | **138** |

**S1-Table 1: Fish individuals included in this study.** Classification is from Nelson, 2016.

**S1-Table 2: Average environmental information on the two sites during sampling**

|  | **Barrier** | | | | **Fringing** | | |
| --- | --- | --- | --- | --- | --- | --- | --- |
|  | **Day 1** | **Day 2** | **Day 3** | **Day 1** | | **Day 2** | **Day 3** |
| Min. depth | 3 | 2 | 2 | 1 | | 0.5 | 0.5 |
| Max. depth | 5 | 5 | 5 | 5 | | 6.5 | 6.5 |
| Swell (0-3) | 1 | 2 | 0.5 | 2 | | 1 | 1 |
| Sunshine (0-3) | 1 | 2.5 | 1 | 1 | | 2.5 | 2 |
| Turbidity (0-3) | 0 | 0 | 0 | 0 | | 0 | 1 |
| Ambient temperature (°C) | 28.9 | 30.5 | 28.6 | 28.6 | | 30.2 | 27.8 |
| Water temperature (°C) | 28.2 | 28.0 | 27.6 | 28.4 | | 29.1 | 28.0 |
| Conductivity (mS) | 47.7 | 49.7 | 49.3 | 50.1 | | 50.0 | 50.3 |
| Salinity (ppt) | 30.9 | 32.5 | 32.2 | 32.7 | | 32.7 | 32.9 |
| TDS (g/L) | 30.9 | 32.3 | 32.0 | 32.5 | | 32.5 | 32.7 |

Minimum and maximum depth were measured using the bathymetric sensor of the boat. Swell, sunshine and sunshine were visually estimated; 0 meaning respectively a calm sea, a sky completely covered by storm clouds and no visibility in the water after 5 meters; 3 meaning respectively an average wave height of 3 meters, a completely clear sky, and a clear water. Ambient and water temperature, conductivity, salinity and Total Dissolved Solids (TDS) were measured using a CO 310 field probe (VWR, USA). All measures were done twice a day, at the beginning (around 10:00 am) and at the end of sampling (around 17:30 pm).

**Supplementary Information S2:**

**
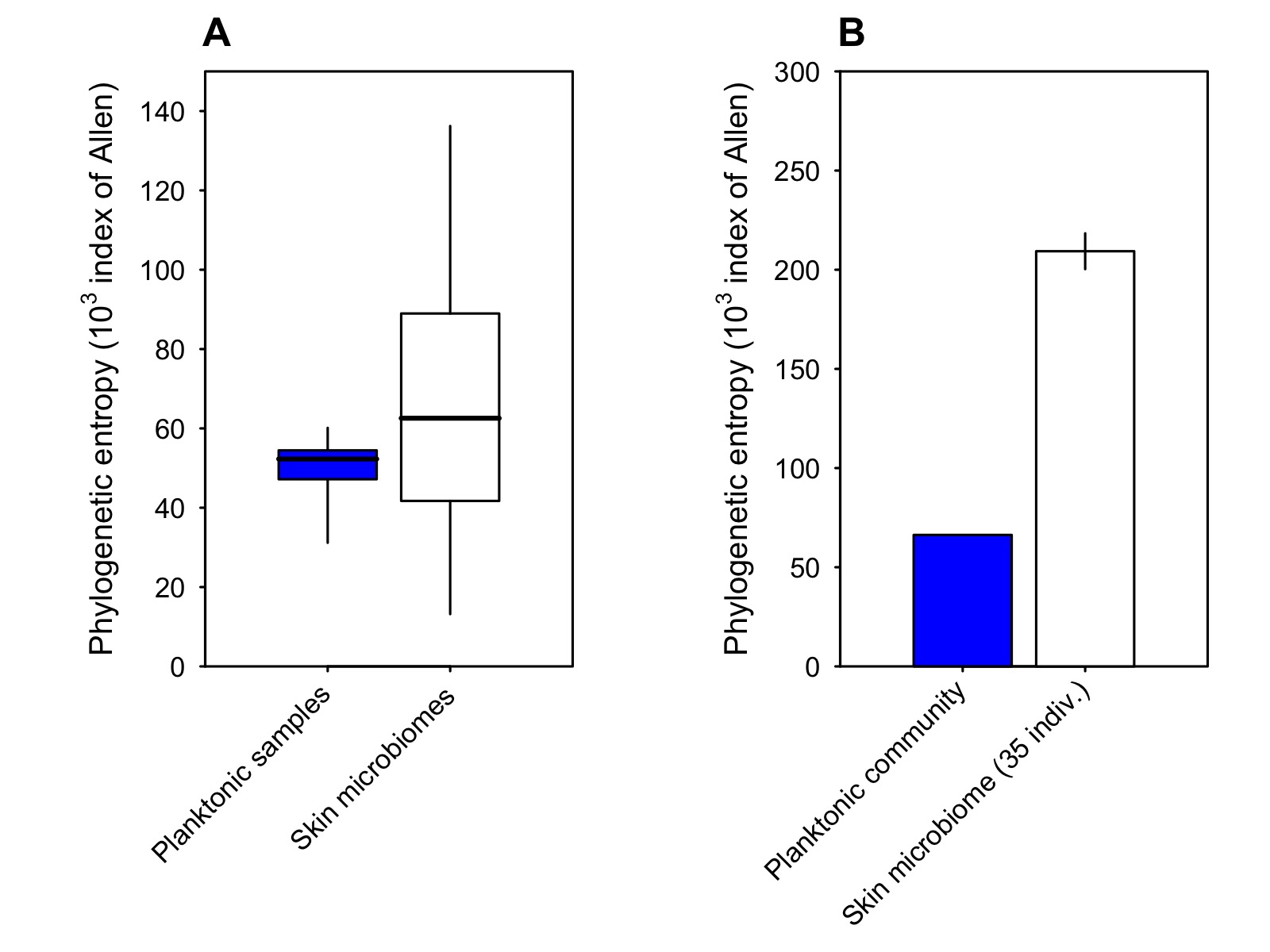
S2-Fig: (A) Phylogenetic entropy of planktonic samples and skin microbiome of each individual and (B) phylogenetic entropy of the whole planktonic community (35 samples) and of 35 randomly subsampled fish microbiomes.** On graph A, thick bars represent the median of phylogenetic entropy values and boxes represent the interquartile range of phylogenetic entropy values. Vertical segments extend to the fifth and the 95^th^ percentiles of values. On graph B, the blue bar represents the phylogenetic entropy of the 35 planktonic samples combined. The white bar represents the phylogenetic entropy found in skin microbiomes of 35 fish individuals (average and associated standard deviation over 100 random subset of individuals).

**Supplementary Information S3:**


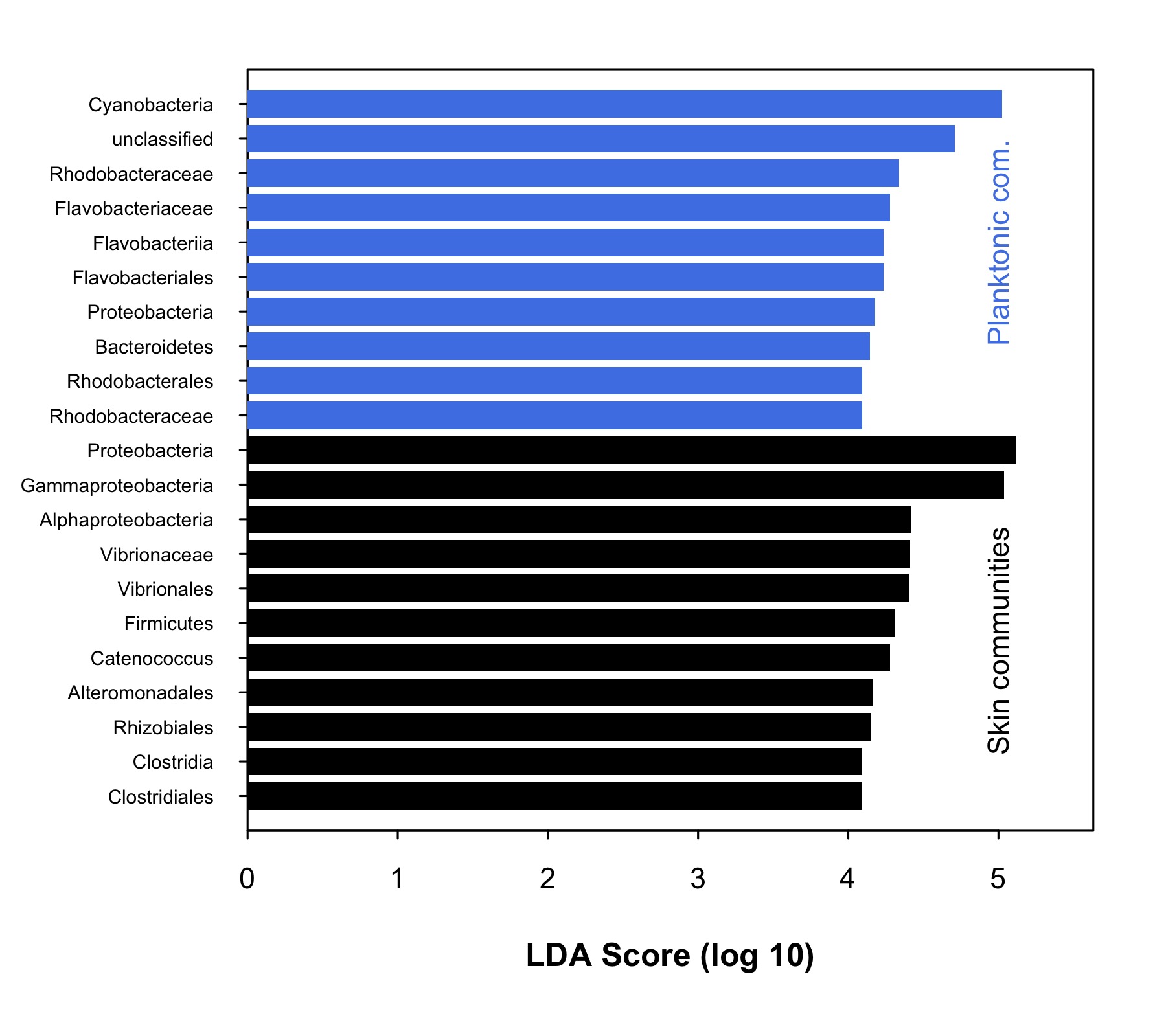


**S3-Fig:** **LefSe analysis showing the most differential microbial taxa** between fish skin-associated and planktonic communities. Only microbial taxa that raised an LDA score > 4 are shown. See supplementary methods for information and references about LefSe.

**S3-Table: Full results of LefSe analysis,** showing all significant biomarkers detected in fish skin microbiome and in planktonic communities

| Prokaryotic clades enriched in fish skin | log10 LDA Score |  | Prokaryotic clades enriched in seawater | log10 LDA Score |
| --- | --- | --- | --- | --- |
| *Proteobacteria* | 5,12 |  | *Cyanobacteria* | 5.02 |
| *Gammaproteobacteria* | 5.04 |  | *unclassified* | 4.71 |
| *Alphaproteobacteria* | 4.42 |  | *Rhodobacteraceae* | 4.34 |
| *Vibrionaceae* | 4.41 |  | *Flavobacteriaceae* | 4.28 |
| *Vibrionales* | 4.41 |  | *Flavobacteriales* | 4.24 |
| *Firmicutes* | 4.31 |  | *Flavobacteriia* | 4.24 |
| *Catenococcus* | 4.28 |  | *Proteobacteria* | 4.18 |
| *Alteromonadales* | 4.17 |  | *Bacteroidetes* | 4.14 |
| *Rhizobiales* | 4.15 |  | *Rhodobacteraceae* | 4.09 |
| *Clostridia* | 4.09 |  | *Rhodobacterales* | 4.09 |
| *Clostridiales* | 4.09 |  | *Aestuariibacter* | 3.88 |
| *Alteromonadales* | 4.00 |  | *Alteromonadaceae* | 3.88 |
| *Methylobacteriaceae* | 3.94 |  | *Rhodospirillaceae* | 3.59 |
| *Methylobacterium* | 3.94 |  | *Rhodospirillales* | 3.35 |
| *Clostridiales* | 3.91 |  | *BD2 7* | 3.04 |
| *Halomonadaceae* | 3.89 |  | *Cellvibrionales* | 3.02 |
| *Oceanospirillales* | 3.89 |  | *OM182 clade* | 2.66 |
| *Betaproteobacteria* | 3.84 |  | *Betaproteobacteria* | 2.38 |
| *Burkholderiales* | 3.82 |  |  |  |
| *Breoghania* | 3.75 |  |  |  |
| *Bradyrhizobiaceae* | 3.73 |  |  |  |
| *Psychrobium* | 3.73 |  |  |  |
| *Shewanellaceae* | 3.73 |  |  |  |
| *Fusobacteriaceae* | 3.70 |  |  |  |
| *Fusobacteriia* | 3.70 |  |  |  |
| *Fusobacteria* | 3.70 |  |  |  |
| *Fusobacteriales* | 3.70 |  |  |  |
| *Aliivibrio* | 3.68 |  |  |  |
| *Ilyobacter* | 3.68 |  |  |  |
| *Firmicutes* | 3.67 |  |  |  |
| *Planctomycetales* | 3.59 |  |  |  |
| *Planctomycetaceae* | 3.59 |  |  |  |
| *Planctomycetes* | 3.59 |  |  |  |
| *Planctomycetacia* | 3.59 |  |  |  |
| *Bacilli* | 3.56 |  |  |  |
| *Campylobacterales* | 3.55 |  |  |  |
| *Epsilonproteobacteria* | 3.55 |  |  |  |
| *Actinobacteria* | 3.54 |  |  |  |
| *Campylobacteraceae* | 3.50 |  |  |  |
| *Nesiotobacter* | 3.50 |  |  |  |
| *Oxalobacteraceae* | 3.43 |  |  |  |
| *Campylobacteraceae* | 3.43 |  |  |  |
| *Bacillales* | 3.37 |  |  |  |
| *Burkholderiales* | 3.33 |  |  |  |
| *Sphingobacteriia* | 3.32 |  |  |  |
| *Sphingobacteriales* | 3.32 |  |  |  |
| *Rhodospirillum* | 3.31 |  |  |  |
| *Planctomycetaceae* | 3.31 |  |  |  |
| *Vibrionaceae* | 3.31 |  |  |  |
| *Comamonadaceae* | 3.31 |  |  |  |
| *Burkholderiales* | 3.30 |  |  |  |
| *Chitinophagaceae* | 3.29 |  |  |  |
| *Hydrotalea* | 3.26 |  |  |  |
| *Azorhizobium* | 3.26 |  |  |  |
| *Rhodospirillales* | 3.23 |  |  |  |
| *Micrococcales* | 3.20 |  |  |  |
| *Bacillales* | 3.17 |  |  |  |
| *Clostridiaceae 1* | 3.13 |  |  |  |
| *Roseimaritima* | 3.13 |  |  |  |
| *FamilyI* | 3.11 |  |  |  |
| *Acetobacteraceae* | 3.07 |  |  |  |
| *Capnocytophaga* | 3.06 |  |  |  |
| *Bacilli* | 3.06 |  |  |  |
| *Flavihumibacter* | 3.05 |  |  |  |
| *Actinobacteria* | 3.04 |  |  |  |
| *Clostridium sensu stricto* | 3.04 |  |  |  |
| *Clostridiaceae 2* | 3.04 |  |  |  |
| *Proteinivorax* | 3.02 |  |  |  |
| *Family XIV* | 3.02 |  |  |  |
| *Bacillaceae* | 2.98 |  |  |  |
| *Methanomicrobiales* | 2.98 |  |  |  |
| *Lachnospiraceae* | 2.96 |  |  |  |
| *Corynebacteriales* | 2.96 |  |  |  |
| *Acetobacteraceae* | 2.95 |  |  |  |
| *Dermabacteraceae* | 2.95 |  |  |  |
| *Ornithobacterium* | 2.94 |  |  |  |
| *Clostridiaceae 1* | 2.93 |  |  |  |
| *Hyphomonadaceae* | 2.93 |  |  |  |
| *Xanthobacteraceae* | 2.93 |  |  |  |
| *Saccharococcus* | 2.93 |  |  |  |
| *Halobacteriales* | 2.92 |  |  |  |
| *Haematobacter* | 2.91 |  |  |  |
| *Caulobacterales* | 2.91 |  |  |  |
| *Lachnospiraceae* | 2.91 |  |  |  |
| *Halobacteriaceae* | 2.90 |  |  |  |
| *Ruminococcaceae* | 2.89 |  |  |  |
| *Halobacteria* | 2.88 |  |  |  |
| *Desulfotignum* | 2.88 |  |  |  |
| *Sulfurovum* | 2.88 |  |  |  |
| *Deltaproteobacteria* | 2.88 |  |  |  |
| *Hyunsoonleella* | 2.87 |  |  |  |
| *Micrococcaceae* | 2.86 |  |  |  |
| *Thaumarchaeota* | 2.86 |  |  |  |
| *Carnobacteriaceae* | 2.85 |  |  |  |
| *Micrococcales* | 2.85 |  |  |  |
| *Micrococcales* | 2.85 |  |  |  |
| *Sungkyunkwania* | 2.85 |  |  |  |
| *Coriobacteriia* | 2.85 |  |  |  |
| *Coriobacteriales* | 2.84 |  |  |  |
| *Planctopirus* | 2.84 |  |  |  |
| *Auritidibacter* | 2.83 |  |  |  |
| *Pibocella* | 2.83 |  |  |  |
| *Coriobacteriaceae* | 2.82 |  |  |  |
| *Lactobacillales* | 2.80 |  |  |  |
| *Clostridium sensu stricto 17* | 2.78 |  |  |  |
| *Telluria* | 2.77 |  |  |  |
| *Sphingomonadales* | 2.77 |  |  |  |
| *Croceicoccus* | 2.77 |  |  |  |
| *Erythrobacteraceae* | 2.77 |  |  |  |
| *Zunongwangia* | 2.74 |  |  |  |
| *Nonlabens* | 2.73 |  |  |  |
| *Bacillaceae* | 2.73 |  |  |  |
| *Helicobacteraceae* | 2.73 |  |  |  |
| *Glaciecola* | 2.72 |  |  |  |
| *Desulfobacteraceae* | 2.71 |  |  |  |
| *DEV007* | 2.71 |  |  |  |
| *Cytophagales* | 2.71 |  |  |  |
| *Cytophagia* | 2.70 |  |  |  |
| *Arcobacter* | 2.70 |  |  |  |
| *Prevotellaceae* | 2.70 |  |  |  |
| *Prevotella 7* | 2.69 |  |  |  |
| *Neisseriaceae* | 2.69 |  |  |  |
| *Stenoxybacter* | 2.69 |  |  |  |
| *Cenarchaeales* | 2.68 |  |  |  |
| *Bacteroidia* | 2.68 |  |  |  |
| *Cenarchaeaceae* | 2.68 |  |  |  |
| *Neisseriales* | 2.68 |  |  |  |
| *Methanomicrobia* | 2.68 |  |  |  |
| *DEV007* | 2.68 |  |  |  |
| *Bacteroidales* | 2.68 |  |  |  |
| *Anaerosporobacter* | 2.67 |  |  |  |
| *Cenarchaeum* | 2.67 |  |  |  |
| *Enterobacteriales* | 2.66 |  |  |  |
| *Cyclobacteriaceae* | 2.66 |  |  |  |
| *Moraxellaceae* | 2.66 |  |  |  |
| *Moraxella* | 2.66 |  |  |  |
| *Pseudomonadales* | 2.66 |  |  |  |
| *Pasteurellaceae* | 2.66 |  |  |  |
| *Solirubrobacterales* | 2.65 |  |  |  |
| *Enterobacteriaceae* | 2.65 |  |  |  |
| *Pasteurellaceae* | 2.65 |  |  |  |
| *Rhodococcus* | 2.65 |  |  |  |
| *Marine Group I* | 2.65 |  |  |  |
| *Corynebacteriales* | 2.64 |  |  |  |
| *Algoriphagus* | 2.64 |  |  |  |
| *Pasteurellales* | 2.63 |  |  |  |
| *Deltaproteobacteria* | 2.63 |  |  |  |
| *Epilithonimonas* | 2.62 |  |  |  |
| *Thermoleophilia* | 2.62 |  |  |  |
| *Nocardiaceae* | 2.60 |  |  |  |
| *Chiayiivirga* | 2.60 |  |  |  |
| *Corynebacterium 1* | 2.58 |  |  |  |
| *Chitinophagaceae* | 2.56 |  |  |  |
| *Thaumarchaeota* | 2.54 |  |  |  |
| *SS1 B 09 64* | 2.54 |  |  |  |
| *Dolosigranulum* | 2.54 |  |  |  |
| *Rickettsiaceae* | 2.53 |  |  |  |
| *Candidatus Cryptoprodotis* | 2.53 |  |  |  |
| *Rickettsiales* | 2.53 |  |  |  |
| *Thaumarchaeota* | 2.53 |  |  |  |
| *Desulfovibrionales* | 2.53 |  |  |  |
| *SS1 B 09 64* | 2.52 |  |  |  |
| *SEEP SRB4* | 2.50 |  |  |  |
| *Chlamydiae* | 2.50 |  |  |  |
| *Chlamydiales* | 2.47 |  |  |  |
| *Simkaniaceae* | 2.47 |  |  |  |
| *Verrucomicrobiales* | 2.47 |  |  |  |
| *Simkania* | 2.46 |  |  |  |
| *Sphingobacteriaceae* | 2.46 |  |  |  |
| *Actinobacteria* | 2.45 |  |  |  |
| *Desulfobulbaceae* | 2.45 |  |  |  |
| *Klugiella* | 2.44 |  |  |  |
| *Verrucomicrobiae* | 2.44 |  |  |  |
| *Microbacteriaceae* | 2.43 |  |  |  |
| *Pedobacter* | 2.42 |  |  |  |
| *Desulfovibrionales* | 2.41 |  |  |  |
| *Rhizobium* | 2.41 |  |  |  |
| *Euryarchaeota* | 2.39 |  |  |  |
| *Rhizobiaceae* | 2.38 |  |  |  |
| *Mollicutes RF9* | 2.38 |  |  |  |
| *Mollicutes* | 2.37 |  |  |  |
| *Thermoplasmata* | 2.37 |  |  |  |
| *Thermoplasmatales* | 2.37 |  |  |  |
| *Thermoplasmatales Incertae Sedis* | 2.37 |  |  |  |
| *Spirochaetales* | 2.36 |  |  |  |
| *Mollicutes RF9* | 2.36 |  |  |  |
| *Spirochaetaceae* | 2.36 |  |  |  |
| *Tenericutes* | 2.36 |  |  |  |
| *Spirochaetes* | 2.36 |  |  |  |
| *Frankiales* | 2.35 |  |  |  |
| *Treponema 2* | 2.35 |  |  |  |
| *Geodermatophilaceae* | 2.34 |  |  |  |
| *Xanthomonadales* | 2.34 |  |  |  |
| *Xanthomonadaceae* | 2.34 |  |  |  |
| *Mollicutes RF9* | 2.33 |  |  |  |
| *Lentisphaeraceae* | 2.30 |  |  |  |
| *Lentisphaera* | 2.28 |  |  |  |
| *Lentisphaerae* | 2.28 |  |  |  |
| *Lentisphaerales* | 2.28 |  |  |  |
| *Lentisphaeria* | 2.27 |  |  |  |
| *Elusimicrobium* | 2.26 |  |  |  |
| *Elusimicrobiales* | 2.26 |  |  |  |
| *Elusimicrobia* | 2.26 |  |  |  |
| *Elusimicrobiaceae* | 2.26 |  |  |  |

**Supplementary Information S4:**

**S4-Table : Effect of fish phylogeny on the diversity of its skin microbiome**

|  | **A. Random subsampling** | | **B. Averaged microbiomes** | |
| --- | --- | --- | --- | --- |
| **Index of alpha-diversity** | **Statistic** | **% of sign. P-values (<0.05)** | **Statistic** | **P-value** |
| **Moran’s *I*** | 0.02±0.02 | 48.8 | 0.02 | 0.05 |
| **Pagel’s Lambda** | 0.41±0.33 | 34.0 | 0.23 | 0.28 |

The effect of fish phylogeny on its skin-associated microbial diversity was assessed using *i)* Moran’s *I* index of autocorrelation scaling between -1 and 1, 1 meaning that there is a strong correlation between phylogeny and the trait value tested; and *ii)* Pagel’s Lambda which equals 0 when there is no phylogenetic signal and equals 1 when trait values conforms predictions of a model of Brownian evolution. Lambda and *I* values were compared to the ones obtained from a null model were diversity values were randomly shuffled on fish phylogeny (999 replicates). In case of method A, Mean and associated standard deviation of Lambda and Moran’s *I* on 999 subsamples are provided, as well as the percentage of subsamples where the correlation was significant (P<0.05). In case of method B, observed Pagel’s Lambda and Moran’s I were indicated, as well as the associated P-value.

**Supplementary Information S5: Sampling sites metadata and effect on fish skin microbial community structure**

**S5-Table 1: Effect of reef type and fish species on fish skin microbial community structure**

|  | **Weighted Unifrac** | |
| --- | --- | --- |
| **Factor** | **P** | **R^2^** |
| Reef type | 0.002 | 0.03 |
| Fish species | 0.001 | 0.32 |
| Reef type * Fish species | 0.026 | 0.19 |

The effect of reef type versus the one of fish species was assessed by computing a permutational multivariate ANOVA (PERMANOVAs) on abundance weighted and unweighted phylogenetic dissimilarities (W-Unifrac and U-Unifrac) between the 74 individuals belonging to the 16 fish species that were sampled on both sites.

**Supplementary Information S6: Results of BDTT (Beta-Diversity Through Time) assessing the correlation between microbiome variability and hosts’ phylogeny across bacterial phylogenetic tree**

|  | **A. Random subsampling** | | **B. Averaged microbiomes** | |
| --- | --- | --- | --- | --- |
| **Time (Mya)** | **% of sign. P-values** | **R^2^** | **P-value** | **R^2^** |
| **0** | 0 | 0.001±0.00 | 0.17 | 0.01 |
| **100** | 0 | 0.001±0.00 | 0.18 | 0.01 |
| **200** | 0 | 0.001±0.00 | 0.18 | 0.1 |
| **300** | 0 | 0.001±0.00 | 0.14 | 0.01 |
| **400** | 0 | 0.001±0.00 | 0.14 | 0.01 |
| **500** | 0 | 0.001±0.00 | 0.10 | 0.01 |
| **600** | 0 | 0.002±0.00 | 0.08 | 0.02 |
| **700** | 0 | 0.002±0.00 | 0.07 | 0.02 |
| **800** | 0 | 0.001±0.00 | 0.08 | 0.02 |
| **900** | 0 | 0.001±0.00 | 0.10 | 0.01 |

This method is from Groussin et al. Nature Communication 2016, (see Supplementary Methods) for 9 periods of 100 millions of years from present time (t=0) to 900 Mya in the past. At each period of time, BDTT computes microbiome variability using Bray-Curtis dissimilarity and assesses its correlation with hosts’ divergence times using a Mantel test. This method was tested A. on W-Unifrac matrices calculated using 999 subsampling replicates of one individual per fish species and B. on W-Unifrac matrices calculated using averaged microbiomes of individuals of each species (see Supplementary Methods for more details). In method A., Mean and associated standard deviation of R-squared values on 999 subsamples are provided, as well as the percentage of subsamples where the correlation was significant (P<0.05).

Results of the Mantel tests show that even when considering deep prokaryotic phylogenetic clades (*i.e.* 900 Mya correspond roughly to the apparition of Bilaterians and divergence of bacterial orders), there is no correlation between host phylogeny and the structure and composition of its associated skin microbiome.

**Supplementary Information S7: Fish ecological traits**


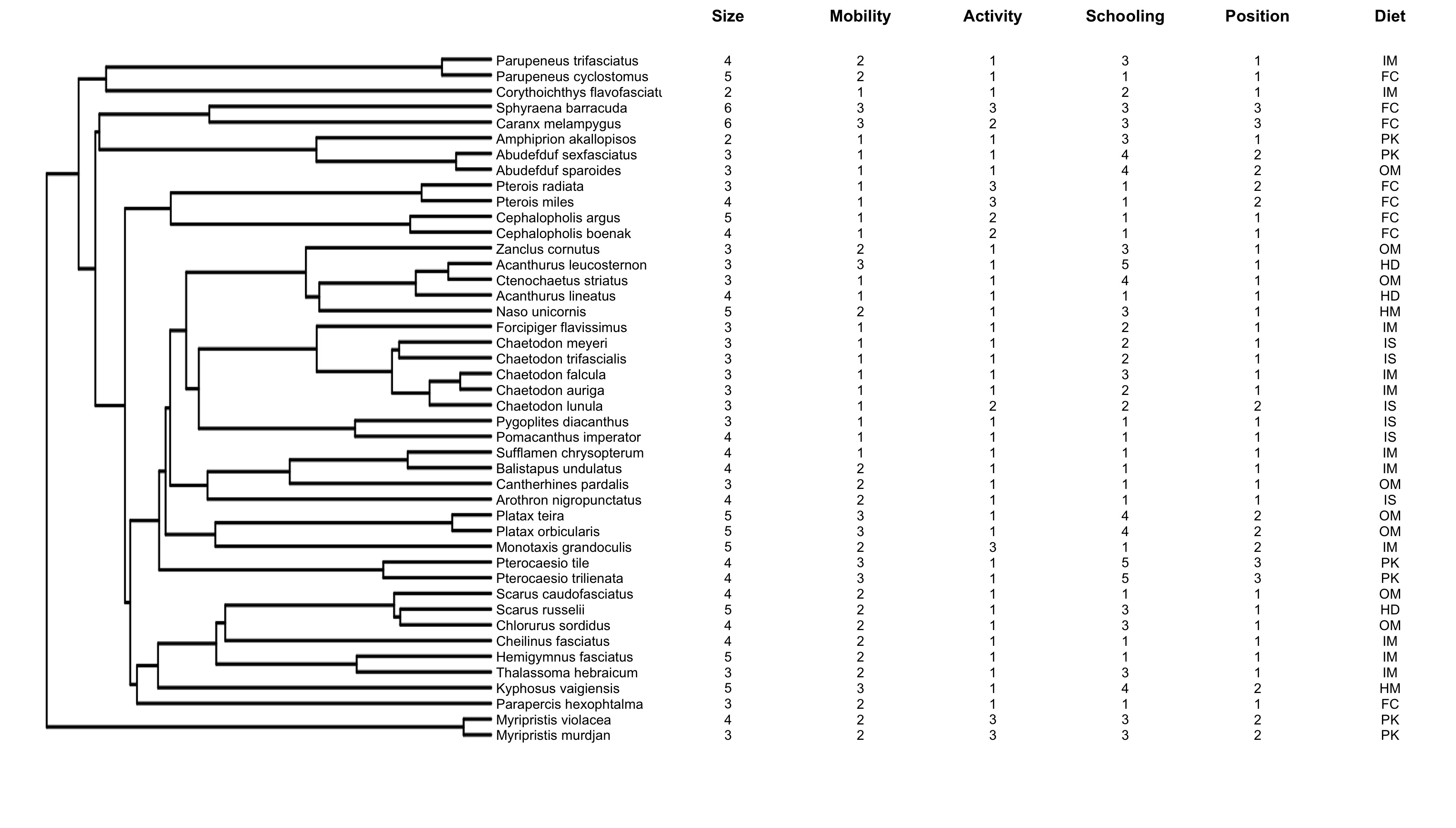


**S7-Figure:** Distribution of functional trait values among fish phylogeny. Traits are encoded as ordinal variables as indicated in S8-Table.

**S7-Table: Encoding of functional traits tested in this study**

| **Functional traits** | **Encoding** |
| --- | --- |
| Size at maturity | **1**: 7.1-15 cm; **2**: 15.1- 30cm; **3**: 30.1-50cm; **4**: 30.1-50cm; **5**: 50.1-80cm; **6**: >80cm |
| Mobility | **1**: sedentary; **2**: mobile within a reef; **3**: highly mobile, *i.e.* between reefs |
| Activity | **1**: diurnal; **2**: diurnal and nocturnal; **3**: nocturnal |
| Schooling | **1**: solitary; **2**: pairing; **3**: small group; **4**: medium group; **5**: large group |
| Position | **1**: sea bottom; **2**: above sea bottom; **3**: pelagic |
| Diet | **FC**: Pelagic macro-organisms, *i.e.* large organisms living in the water column, as well as benthic fishes  **IM**: Mobile invertebrates, *i.e.* all-size mobile benthic invertebrates  **IS**: Sessile invertebrates, *i.e.* corals, sponges, ascidians and all other sessile benthic invertebrates  **PK**: Planktonivorous fishes are fishes eating planktonic and small benthic organisms that can migrate in the water column (copepods, crustacean larvae…)  **HM**: Herbivorous macro-algal fishes are fishes eating macro-algae and sea grass  **HD**: Herbivorous-detritivorous fishes eat undefined or detritical material  **OM**: Omnivorous fishes regroup fishes that are both herbivorous or detritivorous, and carnivorous |

**Supplementary Information S8: Effect of fish ecological traits on their associated skin microbiome**

**S8-Table: Effect of fish ecological traits on the structure of skin microbial communities**

|  | **A. Random subsampling** | | **B. Averaged microbiomes** | |
| --- | --- | --- | --- | --- |
| **Ecological Trait** | **% of sign. P-values** | **Mean R^2^** | **P-value** | **R^2^** |
| **Diet** | **88.1** | **0.18 ± 0.01** | **0.002** | **0.20** |
| Size | 8.8 | 0.10 ± 0.01 | 0.06 | 0.11 |
| Schooling | 24.4 | 0.10 ± 0.01 | 0.12 | 0.10 |
| Mobility | 2.1 | 0.05 ± 0.01 | 0.40 | 0.04 |
| Position | 4.6 | 0.04 ± 0.01 | 0.76 | 0.04 |
| Activity | 0.1 | 0.04 ± 0.01 | 0.99 | 0.03 |

Results of the PERMANOVAs-based analysis assessing the effect of fish ecological traits A. performed on W-Unifrac matrices calculated using 999 subsampling replicates of one individual per fish species and B. performed on W-Unifrac matrices calculated using averaged microbiomes of individuals of each species (see Supplementary Methods for more details). In method A., Mean and associated standard deviation of R-squared values on 999 subsamples are provided, as well as the percentage of subsamples where the correlation was significant (P<0.05).


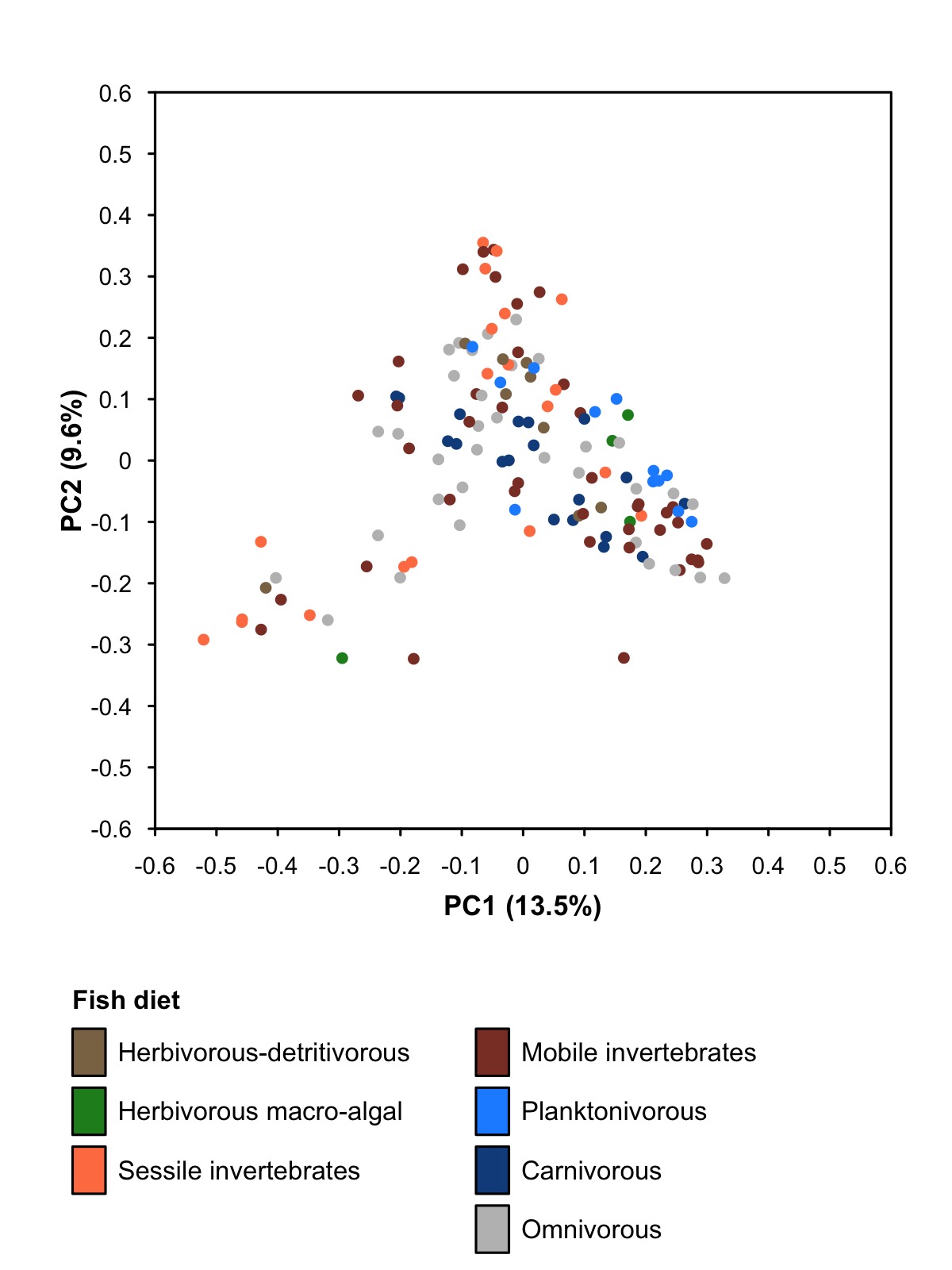


**S8-Fig: PCoA plot** representing all individuals included in this study, based on abundance weighted-Unifrac dissimilarity values (W-Unifrac) between microbiomes. Individuals were colored according to their main prey.

**Supplementary Information S9: Investigation of an eventual transfer from sessile invertebrates-associated cells to fish skin microbiome**

At the same time of sampling and on the same sites, 83 sessile invertebrates were sampled, representing a wide range of clades, *i.e.* scleratinian corals, soft corals, sponges, anemones and gorgonians. Surface microbiome was sampled, either by sampling of surface mucus, or swabbing, and was analyzed using the same methods as for fish skin swabs. We computed the phylogenetic dissimilarity between surface microbiome of these sessile organisms, and fish skin surface microbiome, depending on fish diet.


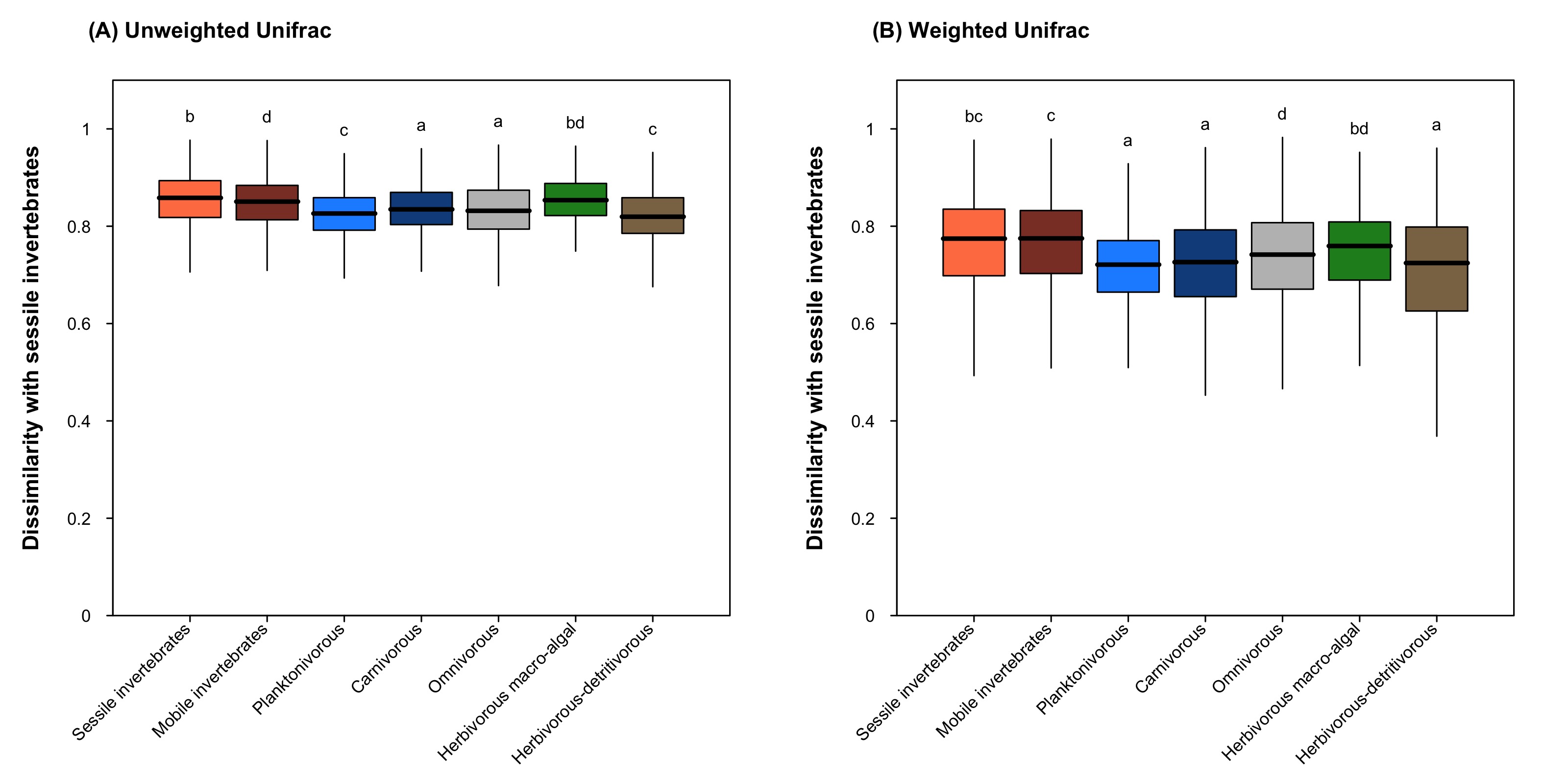
**S9-Figure : Weighted-Unifrac (B) dissimilarity values between sessile invertebrates’ microbiomes and fish skin microbiomes**, separated by fish diets. Phylogenetic dissimilarities were significantly different between different fish diets (Kruskal-Wallis test, P<0.001). Post-hoc pairwise comparisons of dissimilarities among different diets are represented by letters, two different letters indicating a significant difference between dissimilarity values between diets (post-hoc pairwise comparisons available in *pgirmess* package, P<0.05). Fishes eating sessile invertebrates hosted a microbiome not significantly closer to the one of sessile invertebrates, than fishes having contrasting diets.

**Supplementary Information S10: Core microbiome of each fish species**

**S10-Table: OTUs recovered in all sampled individuals of each species**

|  | **Nb ind.** | **Nb core OTUs** | **Cum. rel. abundance (%)** | **Taxonomy of major OTUs** |
| --- | --- | --- | --- | --- |
| *Abudefduf sexfasciatus* | 3 | 14 | 48.9 | *Methylobacterium, Bradyrhizobium, Catenococcus* and *Alphaproteobacteria* |
| *Abudefduf sparoides* | 4 | 2 | 6.4 | *Alteromonadales* and *Alphaproteobacteria* |
| *Acanthurus leucosternon* | 5 | 4 | 1.2 | *Rhodospirillaceae, Rhidobacteraceae, unclass. Gammaproteobacteria* |
| *Acanthurus lineatus* | 3 | 26 | 57.9 | *Haematobacter, Methylobacterium, Rubritaela, unclass. Cyanobacteria, Alpha- et Gammaproteobacteria* |
| *Amphiprion akallopisos* | 3 | 25 | 40 | *Aestuariibacter, Catenococcus, Oceanospirillales, SAR11 and Alteromonadales* |
| *Balistapus undulatus* | 3 | 16 | 42.1 | *Peredibacter, unclass. Alpha-, Gammaproteobacteria, Parcubacteria and Gracilibacteria* |
| *Caranx melampygus* | 3 | 23 | 32.8 | *Aestuariibacter, Halomonadaceae, Rhodobacteraceae, Sphingobacteriales and unclass. Gammaproteobacteria* |
| *Cephalopholis argus* | 6 | 6 | 5.7 | *Phycisphaeraceae, Catenococcus and Rhodobacteraceae* |
| *Chaetodon falcula* | 6 | 0 | 0 | *-* |
| *Chaetodon lunula* | 4 | 4 | 20.4 | *Alistipes, Erysipelotrichales, Bacteroidiales and unclassified Gammaproteobacteria* |
| *Chaetodon meyeri* | 3 | 23 | 36.9 | *Methylobacterium, Bradyrhizobium, Rhodospirillaceae and unclass. Cyanobacteria and Gammaproteobacteria* |
| *Chaetodon trifascialis* | 3 | 13 | 39.9 | *Methylobacterium, Bradyrhizobium, and unclassified Gammaproteobacteria* |
| *Cheilinus fasciatus* | 3 | 40 | 55.3 | *Catenococcus, Aestuariibacter, Halomonadaceae, Flavobacteriaceae, Alteromonadales and unclass. Gammaproteobacteria* |
| *Chlorurus sordidus* | 6 | 10 | 5 | *Bradyrhizobium, Rhodobacteraceae, Thiotrichales and unclas. Alpha- and Gammaproteobacteria* |
| *Corythoichthys flavofasciatus* | 3 | 40 | 70.3 | *Roseibacillus, Rhodobacteraceae and unclassified Cyanobacteria and Alphaproteobacteria* |
| *Ctenochaetus striatus* | 7 | 3 | 8.9 | *Catenococcus, Rhodobacteraceae, Flavobacteriaceae* |
| *Forcipiger flavissimus* | 3 | 7 | 3.2 | *Hydrotalea, Halomonadaceae and unclassified Alpha- and Gammaproteobacteria* |
| *Hemigymnus fasciatus* | 3 | 17 | 52.8 | *Catenococcus, Aestuariibacter and unclassified Gammaproteobacteria* |
| *Kyphosus vaigiensis* | 3 | 27 | 55.4 | *Aestuariibacter, Catenococcus, Methylobacterium, Alteromonadales and unclassified Gammaproteobacteria and Gracilibacteria* |
| *Monotaxis grandoculis* | 5 | 9 | 35.4 | *Aestuariibacter, Halomonadaceae, Alteromonadales, Oceanospirillales and unclass. Gammaproteobacteria* |
| *Parapercis hexophtalma* | 3 | 23 | 44.4 | *Catenococcus, Rhodobacteraceae and unclass. Alpha- and Gammaproteobacteria* |
| *Parupeneus trifasciatus* | 3 | 14 | 76.8 | *Nesiotobacter, Phycisphaeraceae, Flammeovirgaceae and unclass. Gammaproteobacteria* |
| *Platax orbicularis* | 5 | 0 | 0 | *-* |
| *Pomacanthus imperator* | 3 | 110 | 51.4 | *Odoribacter, Rikenella, Victivallaceae, Ruminococcaceae* |
| *Pygoplites diacanthus* | 6 | 2 | 32.6 | *Breoghania, Peregrinibacteria* |
| *Scarus caudofasciatus* | 4 | 6 | 2.3 | *Flavobacteriaceae, Vibrionaceae and unclass. Alpha- and Gammaproteobacteria* |
| *Sufflamen chrysopterum* | 6 | 2 | 1.3 | *Ilyobacter, Gracilibacteria* |
| *Thalassoma hebraicum* | 3 | 22 | 46.5 | *Methylobacterium, Aestuariibacter, Alteromonadales and unclass. Alpha- and Gammaproteobacteria* |
| *Zanclus cornutus* | 6 | 2 | 3.3 | *Clostridiales and unclassified Proteobacteria* |

For each of the 29 species represented by at least three individuals, prokaryotic core OTUs were defined as the OTUs that were recovered in all individuals sampled of a given species. The cumulative relative abundance of such OTUs is indicated, as well as the finest phylogenetic affiliation of the 5 most abundant core OTUs of each species.

**
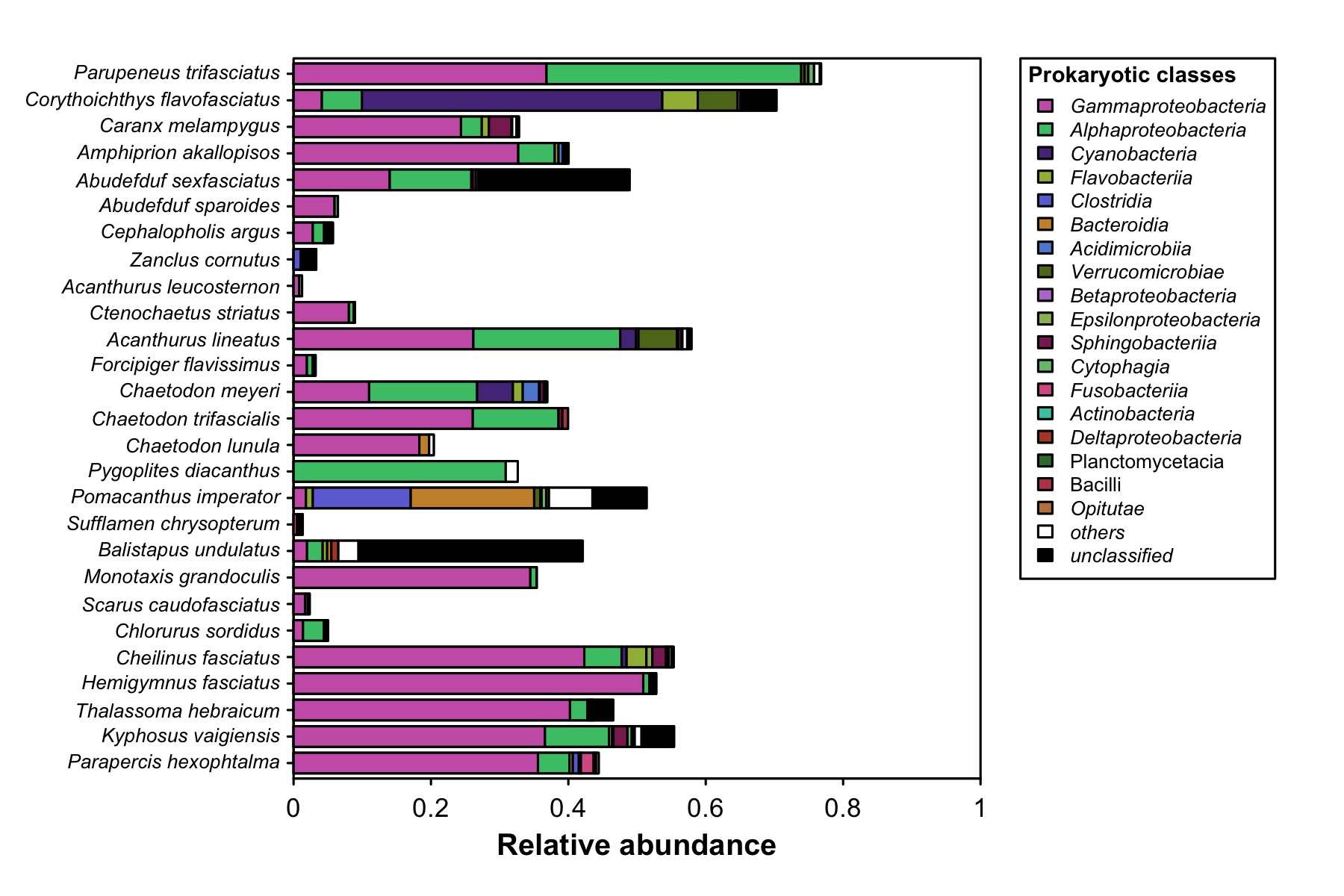
**

**S10-Figure:** Mean class-level composition of fish skin core OTUs. The 18 most abundant classes in all communities are represented with different colors, and other classes are in white.

**Supplementary Information S11: Rarefaction curves**


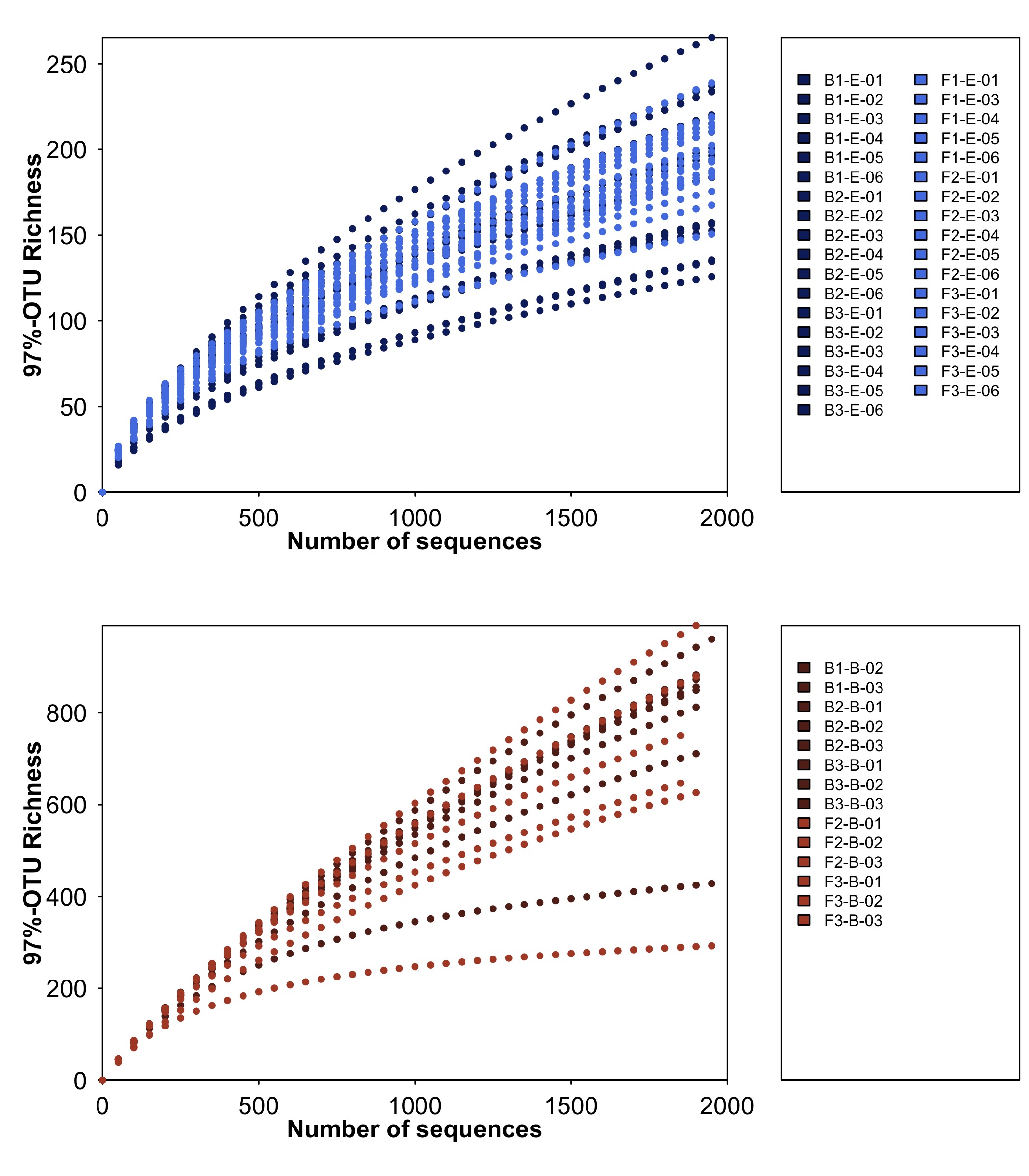


**S11-Fig1:** Rarefaction curves obtained from each planktonic community. Sample names starting by ‘B-‘ were taken on barrier reef, while sample names starting by ‘F-’ were taken on fringing reef.


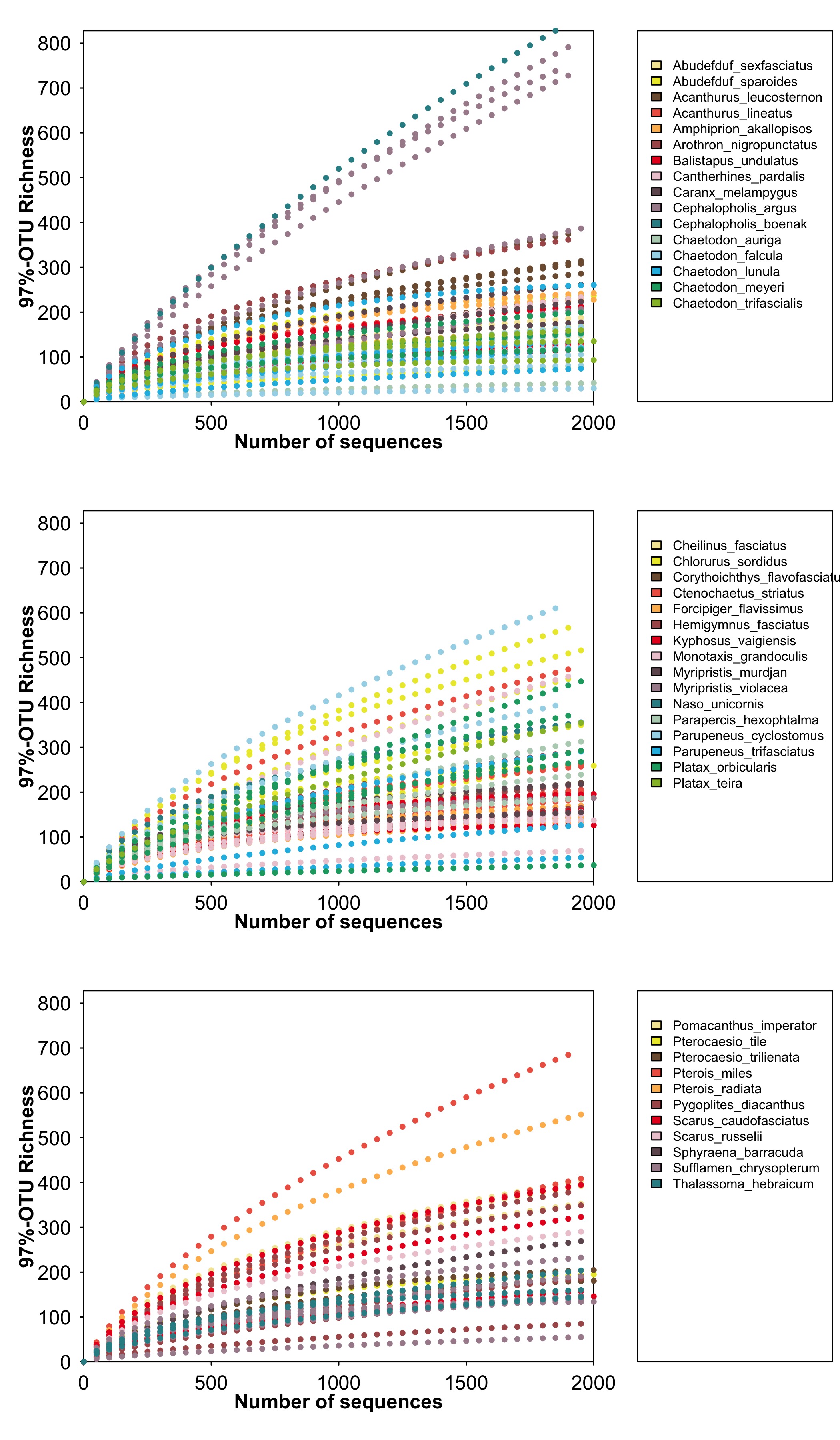


**S11-Fig2: Rarefaction curves for all fish individuals**. The most undersampled species (showing a non-flat curve) were also the phylogenetically richer ones (*Pterois miles*, *Pterois radiata*, *Parupeneus cyclostomus*, *Chlorurus sordidus* and *Cephalophilis boenak*, see Fig 1).

**Supplementary Information S12:**

**
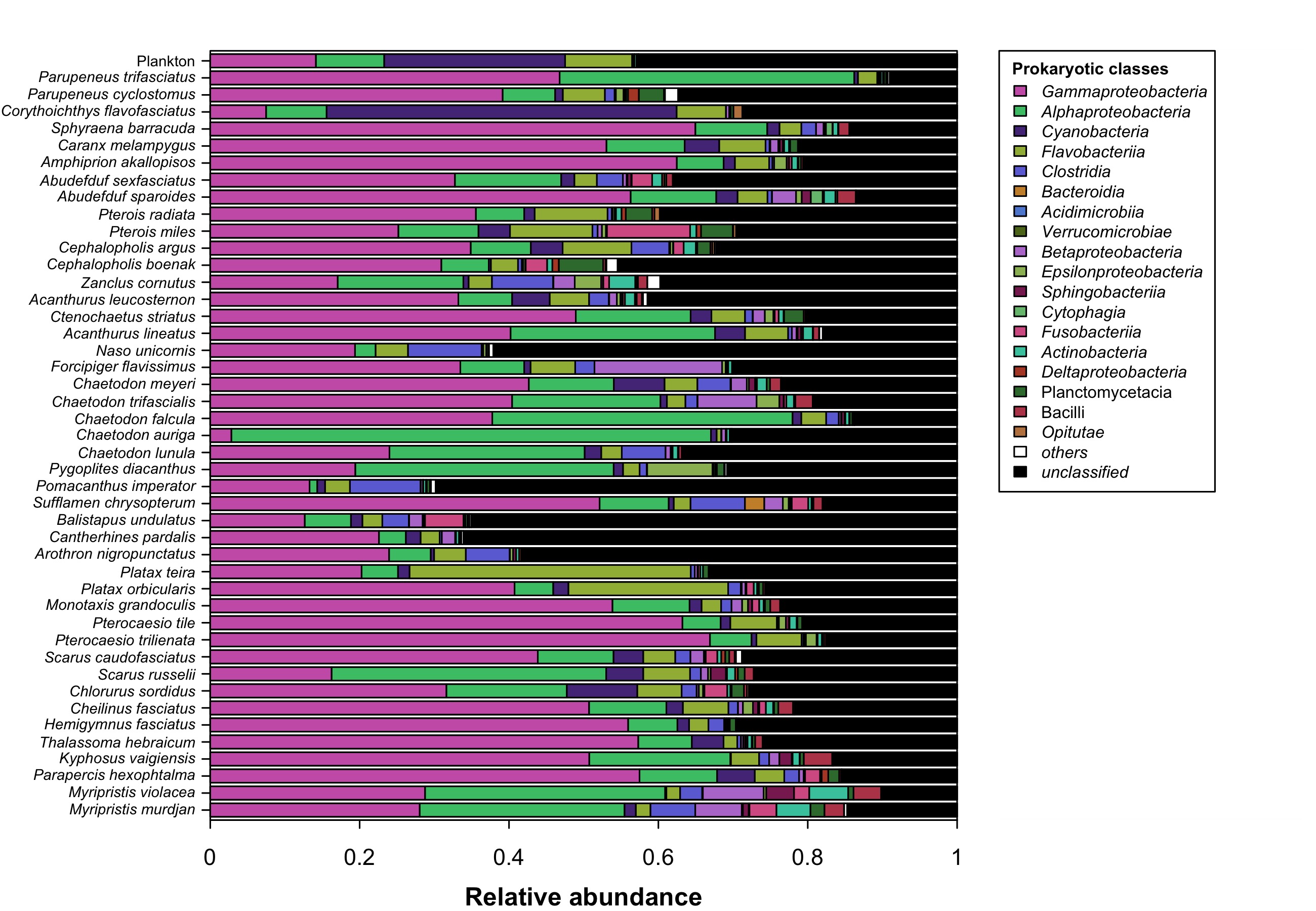
**

**S12-Fig**: Mean class-level composition of fish skin microbiomes and planktonic communities, according to Mothur software. The 18 most abundant classes in all communities are represented with different colors, and other classes are in white. The mean composition of planktonic communities is indicated at the top.

| **Kingdom** | **Phylum** | **Class** | **%of uncl. OTUs** | **Range of rel. abundance (%)** | **Range of number of hosts** |
| --- | --- | --- | --- | --- | --- |
| *Bacteria* | *Verrucomicrobia* | *Verrucomicrobiae* | 7.4 | 0-19.0 | 4-53 |
|  |  | *WCHB1-41** | 1.1 | 0-10.2 | 5-37 |
|  |  | *R76-B128** | 0.4 | 0-0.6 | 14-45 |
|  |  | *Opitutae* | 0.5 | 0-4.1 | 1-5 |
|  | *Bacteroidetes* | *Bacteroidia** | 19.8 | 0-7.9 | 1-38 |
|  |  | *Cytophagia* | 5.8 | 0-12.7 | 1-88 |
|  |  | *Sphingobacteriia* | 5.6 | 0-34.1 | 2-53 |
|  |  | *Flavobacteriia* | 0.9 | 0-10.3 | 3-54 |
|  |  | *Bacteroidetes Incertae Sedis** | 0.4 | 0-0.3 | 5-5 |
|  |  | *ML602M-17** | 0.2 | 0-0.7 | 9 |
|  | *Firmicutes* | *Clostridia* | 10.9 | 0-4.2 | 1-28 |
|  |  | *Erysipelotrichia** | 0.9 | 0-3.1 | 5-22 |
|  |  | *Negativicutes* | 0.2 | 0-0.3 | 6 |
|  | *Proteobacteria* | *Alphaproteobacteria* | 3.2 | 0-12.6 | 1-85 |
|  |  | *Betaproteobacteria* | 0.2 | 0-4.1 | 15 |
|  |  | *Deltaproteobacteria* | 8.8 | 0-7.1 | 2-57 |
|  |  | *Epsilonproteobacteria* | 0.4 | 0-18.7 | 14-15 |
|  |  | *Gammaproteobacteria* | 1.9 | 0-7.7 | 1-82 |
|  | *Cyanobacteria* | *Chloroplast* | 4.6 | 0-16.5 | 1-61 |
|  |  | *Cyanobacteria* | 1.8 | 0-2.3 | 4-50 |
|  |  | *Melainabacteria** | 3.2 | 0-1.7 | 5-16 |
|  |  | *ML635J-21** | 0.4 | 0-0.9 | 11-20 |
|  | *Planctomycetes* | *Phycisphaerae** | 1.1 | 0-1.7 | 5-110 |
|  |  | *OM190** | 1.2 | 0-0.8 | 5-33 |
|  |  | *vadinHA49** | 0.7 | 0-0.7 | 5-22 |
|  |  | *028H05-P-BN-P5** | 0.2 | 0-1.1 | 5 |
|  |  | *Planctomycetacia* | 0.4 | 0-0.3 | 5-5 |
|  | *Chloroflexi* | *SAR202 clade** | 0.4 | 0-0.1 | 6-7 |
|  |  | *Caldilineae** | 0.4 | 0-0.5 | 5-6 |
|  |  | *KD4-96** | 0.2 | 0-0.2 | 8 |
|  |  | *Anaerolineae** | 0.7 | 0-1.1 | 4-9 |
|  | *Acidobacteria* | *Subgroup 9** | 0.2 | 0-0.7 | 13 |
|  |  | *Subgroup 6** | 0.2 | 0-0.3 | 8 |
|  |  | *Subgroup 26** | 0.2 | 0-0.5 | 7 |
|  |  | *Holophagae** | 0.5 | 0-0.2 | 5-8 |
|  |  | *Blastocatellia** | 0.2 | 0-0.3 | 5 |
|  | *Actinobacteria* | *Acidimicrobiia** | 1.4 | 0-11.0 | 5-85 |
|  |  | *Thermoleophilia* | 0.2 | 0-0.2 | 9 |
|  | *Lentisphaerae* | *Oligosphaeria** | 1.8 | 0-10.9 | 3-28 |
|  |  | *Lentisphaeria* | 1.4 | 0-8.1 | 3-13 |
|  | *Gemmatimonaetes* | *BD2-11 terrestrial group** | 0.4 | 0-0.2 | 6-8 |
|  | *Parcubacteria* | *Candidatus Campbellbacteria** | 0.7 | 0-29.3 | 2-19 |
|  | *Tenericutes* | *Mollicutes* | 1.4 | 0-3.2 | 1-11 |
|  | *Deinococcus-Thermus* | *Deinococci** | 0.2 | 0-0.1 | 9 |
|  | *Spirochaetae* | *Spirochaetes* | 1.6 | 0-2.4 | 1-10 |
|  | *Synergistetes* | *Synergistia** | 0.7 | 0-3.7 | 4-11 |
|  | *Peregrinibacteria* | *Candidatus Peribacteria** | 0.4 | 0-4.4 | 3-7 |
|  | *Chlorobi* | *Chlorobia** | 0.2 | 0-0.1 | 5 |
|  | *Nitrospirae* | *Nitrospira* | 0.2 | 0-0.4 | 6 |
|  | All phyla | *Unclassified* | 3.7 | 0-12.3 | 1-75 |
| *Archaea* | *Euryarchaeota* | *Thermoplasmata* | 1.4 | 0-1.4 | 8-73 |

**S12-Table: Class-level putative affiliation of the most common unclassified OTUs** (unclassified OTUs at phylum level, found in at least 5 samples and/or making more than 1% of abundance in at least one sample) found using Arb parsimony insertion tool. %of uncl. OTUs: Percentage of the selected OTUs belonging to a given class. Range of rel. abundance (%) is the minimum and the maximum relative abundance values of OTU(s) belonging to this class. Range of number of hosts is the minimum and the maximum number of individuals where OTUs belonging to this class were recovered. Single values in this last column indicate the number of individual where the only OTU belonging to this class was recovered. *Asterisks indicate classes that were initially not identified by Mothur in the whole dataset.

**Supplementary Information S13: Host phylogenetic tree and branching of species not initially included in Rabosky et al. (2013) phylogenetic tree**

1. **Rabosky et al. (2013) modified phylogenetic tree with added species in Newick format**


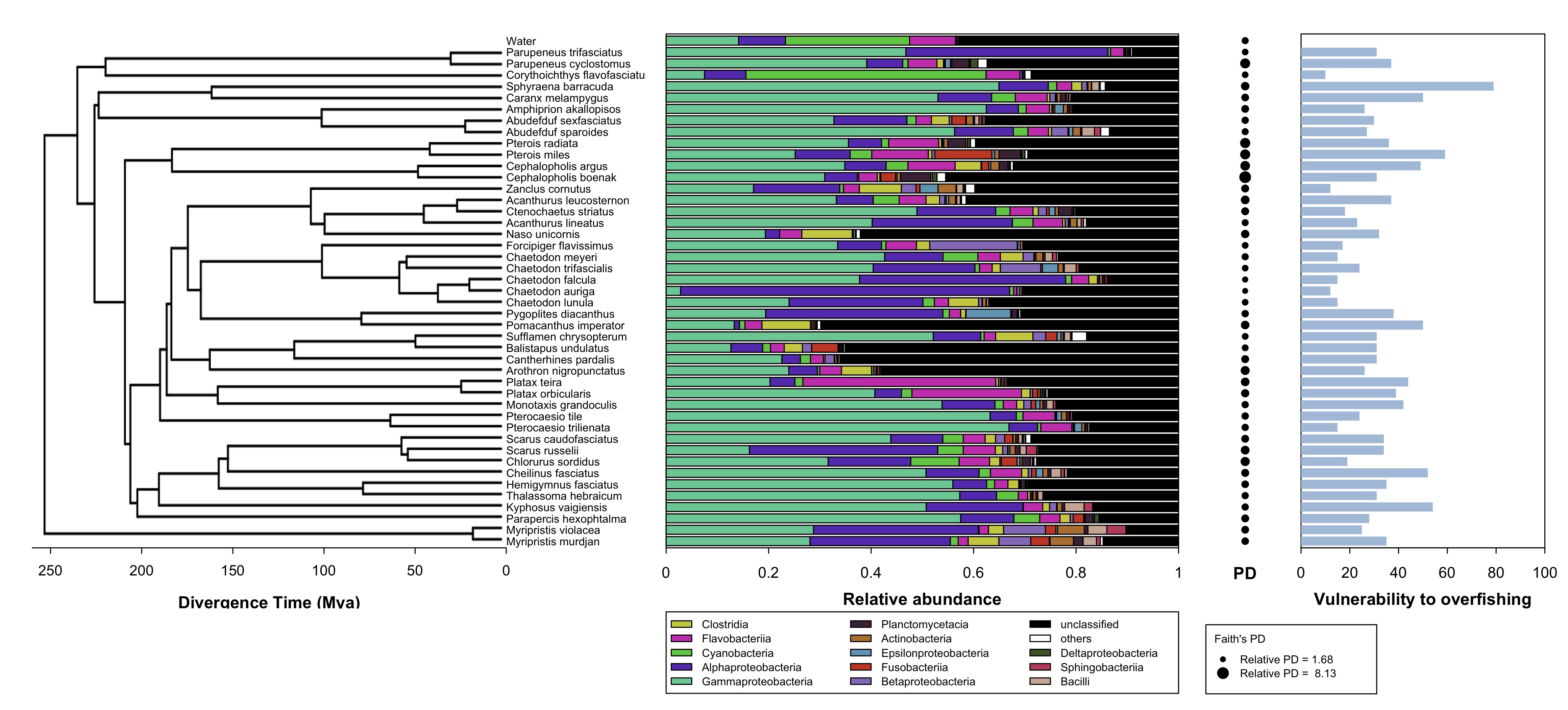


((Myripristis murdjan:8.00651,Myripristis violacea:8.00651)Node3:121.29586,(((((Parapercis hexophtalma:103.0273727,(Kyphosus vaigiensis:96.902137,((Thalassoma hebraicum:39.122854,Hemigymnus fasciatus:39.12286)Node11:40.852082,(Cheilinus fasciatus:77.37268,((Chlorurus sordidus:26.44134,Scarus russelii:26.44134)Node15:1.796755,Scarus caudofasciatus:28.23811)Node14:49.13474)Node12:2.60219)Node10:16.92714)Node9:6.12525)Node8:1.96541,((Pterocaesio trilienata:31.385374,Pterocaesio tile:31.3854)Node17:65.2044,((Monotaxis grandoculis:80.2476,(Platax orbicularis:11.3302,Platax teira:11.33021)Node21:68.91726)Node20:14.4565,((Arothron nigropunctatus:82.517345,(Cantherhines pardalis:58.59547,(Balistapus undulatus:24.27565,Sufflamen chrysopterum:24.275687)Node25:34.3198)Node24:23.92198)Node23:10.871422,(((Pomacanthus imperator:39.592969,Pygoplites diacanthus:39.593)Node28:45.4561,(((Chaetodon lunula:17.92216,(Chaetodon auriga:9.00917,Chaetodon falcula:9.009155)Node32:8.91303)Node31:10.92798,(Chaetodon trifascialis:26.77443,Chaetodon meyeri:26.77445)Node33:2.07571)Node30:21.89785,Forcipiger flavissimus:50.74797)Node29:34.30103)Node27:3.6752,((Naso unicornis:49.999943,(Acanthurus lineatus:21.92091,(Ctenochaetus striatus:12.48236,Acanthurus leucosternon:12.4823649)Node37:9.438535)Node36:28.07914)Node35:3.90017,Zanclus cornutus:53.9002)Node34:34.82407)Node26:4.66462)Node22:1.31527)Node19:1.88563)Node16:8.40301)Node7:1.54063,((Cephalopholis boenak:23.55669,Cephalopholis argus:23.5566)Node39:69.652503,(Pterois miles:20.23159,Pterois radiata:20.23159)Node40:72.97738)Node38:13.3243)Node6:8.62173,(((Abudefduf sparoides:10.19924,Abudefduf sexfasciatus:10.19924)Node43:40.640407,Amphiprion akallopisos:50.839627)Node42:63.144626,(Caranx melampygus:81.9976,Sphyraena barracuda:81.99769)Node44:31.98673)Node41:1.17081)Node5:4.85771,(Corythoichthys flavofasciatus:112.0186,(Parupeneus cyclostomus:14.29613,Parupeneus trifasciatus:14.29613)Node46:97.722467)Node45:7.9941602)Node4:9.2895)Node1;

1. **Branching information of added species and references**

13 species were not initially present in Rabosky et al. phylogenetic tree, and one (*Cephalophilis argus*) was incorrectly branched between *Scaridae* and *Caesionidae*. To define the branching emplacement of these species, we searched for resolved phylogenies of fish families, and searched for the species’ closest relative already included in Rabosky’s tree. We then placed our species in replacement to its closest relative. When possible, we favored studies using genetic data based on multiple loci. No published phylogenetic tree of *Caesionidae* and *Mullidae* based on genetic data could be found; therefore we used studies based on morphological and anatomical traits:

| **Species added** | **Branching emplacement** | **Type of data** | **Reference** |
| --- | --- | --- | --- |
| *Acanthurus leucosternon* | In replacement of *Acanthurus nigricans* | Genetic | (Sorenson et al. 2013) |
| *Acanthurus lineatus* | On a separated branch, connected to the common node of all other *Acanthurus sp.* | Genetic | (Sorenson et al. 2013) |
| *Caesio xanthonota* | In replacement of *Caesio caerulaurea* | Morpho-anatomy | (Carpenter 1990) |
| *Cantherhines pardalis* | In replacement of *Cantherinhes pullus* | Genetic | (Santini, Sorenson, and Alfaro 2013) |
| *Corythoichthys flavofasciatus* | In replacement of *Corythoichthys intestinalis* | No data* |  |
| *Cephalopholis argus* | In replacement of *Cephalopholis nigri* | Genetic | (Craig and Hastings 2007) |
| *Myripristis murdjan* | Branched to the node separating *Myripristis violacea* from all other species | Genetic | (Dornburg et al. 2012) |
| *Parapercis hexophtalma* | In replacement of *Parapercis clathrata* | No data* |  |
| *Parupeneus cyclostomus* | On a separated branch, connected to the common node of all *Upeneichthys sp.* | Morpho-anatomy | (Kim 2002) |
| *Parupeneus trifasciatus* | Branched to the node separating *Parupeneus cyclostomus* from all other species | Morpho-anatomy | (Kim 2002) |
| *Pterocaesio tile* | In replacement of *Pterocaesio digramma* | Morpho-anatomy | (Carpenter 1990) |
| *Pterocaesio trillienata* | In replacement of *Pterocaesio marri* | Morpho-anatomy | (Carpenter 1990) |
| *Scarus caudofasciatus* | In replacement of *Scarus spinus* | Genetic | (Choat et al. 2012) |
| *Scarus russelii* | In replacement of *Scarus flavipectoralis* | Genetic | (Choat et al. 2012) |

*To our knowledge, no phylogenetic tree of *Syngnathidae* including *Corythoichthys flavofasciatus* has been published yet. Therefore, we placed our species in replacement of one of its congenerics. The same problem appeared for *Parapercis hexophtalma*, which was placed in replacement of its only congeneric that also occurs in Indian ocean (Taquet and Diringer 2007)

1. **References**

Carpenter, Kent E. 1990. “A Phylogenetic Analysis of the Caesionidae (Perciformes: Lutjanoidea).” *Copeia* 1990 (3): 692–717. doi:10.2307/1446436.

Choat, John. H., Oya. S. Klanten, Lynne Van Herwerden, D. Ross Robertson, and Kendall D. Clements. 2012. “Patterns and Processes in the Evolutionary History of Parrotfishes (Family Labridae).” *Biological Journal of the Linnean Society* 107 (3): 529–57. doi:10.1111/j.1095-8312.2012.01959.x.

Craig, Matthew T., and Philip A. Hastings. 2007. “A Molecular Phylogeny of the Groupers of the Subfamily Epinephelinae (Serranidae) with a Revised Classification of the Epinephelini.” *Ichthyological Research* 54 (1): 1–17. doi:10.1007/s10228-006-0367-x.

Dornburg, Alex, Jon A. Moore, Rachel Webster, Dan L. Warren, Matthew C. Brandley, Teresa L. Iglesias, Peter C. Wainwright, and Thomas J. Near. 2012. “Molecular Phylogenetics of Squirrelfishes and Soldierfishes (Teleostei: Beryciformes: Holocentridae): Reconciling More than 100 Years of Taxonomic Confusion.” *Molecular Phylogenetics and Evolution* 65 (2): 727–38. doi:10.1016/j.ympev.2012.07.020.

Kim, B. J. 2002. “Comparative Anatomy and Phylogeny of the Family Mullidae (Teleostei: Perciformes).” *Memoirs of the Graduate School of Fisheries Sciences, Hokkaido University (Japan).* http://agris.fao.org/agris-search/search.do?recordID=JP2003001510.

Rabosky, Daniel L., Francesco Santini, Jonathan Eastman, Stephen A. Smith, Brian Sidlauskas, Jonathan Chang, and Michael E. Alfaro. 2013. “Rates of Speciation and Morphological Evolution Are Correlated across the Largest Vertebrate Radiation.” *Nature Communications* 4 (June): ncomms2958. doi:10.1038/ncomms2958.

Santini, Francesco, Laurie Sorenson, and Michael E. Alfaro. 2013. “A New Multi-Locus Timescale Reveals the Evolutionary Basis of Diversity Patterns in Triggerfishes and Filefishes (Balistidae, Monacanthidae; Tetraodontiformes).” *Molecular Phylogenetics and Evolution* 69 (1): 165–76. doi:10.1016/j.ympev.2013.05.015.

Sorenson, Laurie, Francesco Santini, Giorgio Carnevale, and Michael E. Alfaro. 2013. “A Multi-Locus Timetree of Surgeonfishes (Acanthuridae, Percomorpha), with Revised Family Taxonomy.” *Molecular Phylogenetics and Evolution* 68 (1): 150–60. doi:10.1016/j.ympev.2013.03.014.

Taquet, Marc, and Alain Diringer. 2007. *Poissons de l’océan Indien et de la mer Rouge*. Editions Quae.


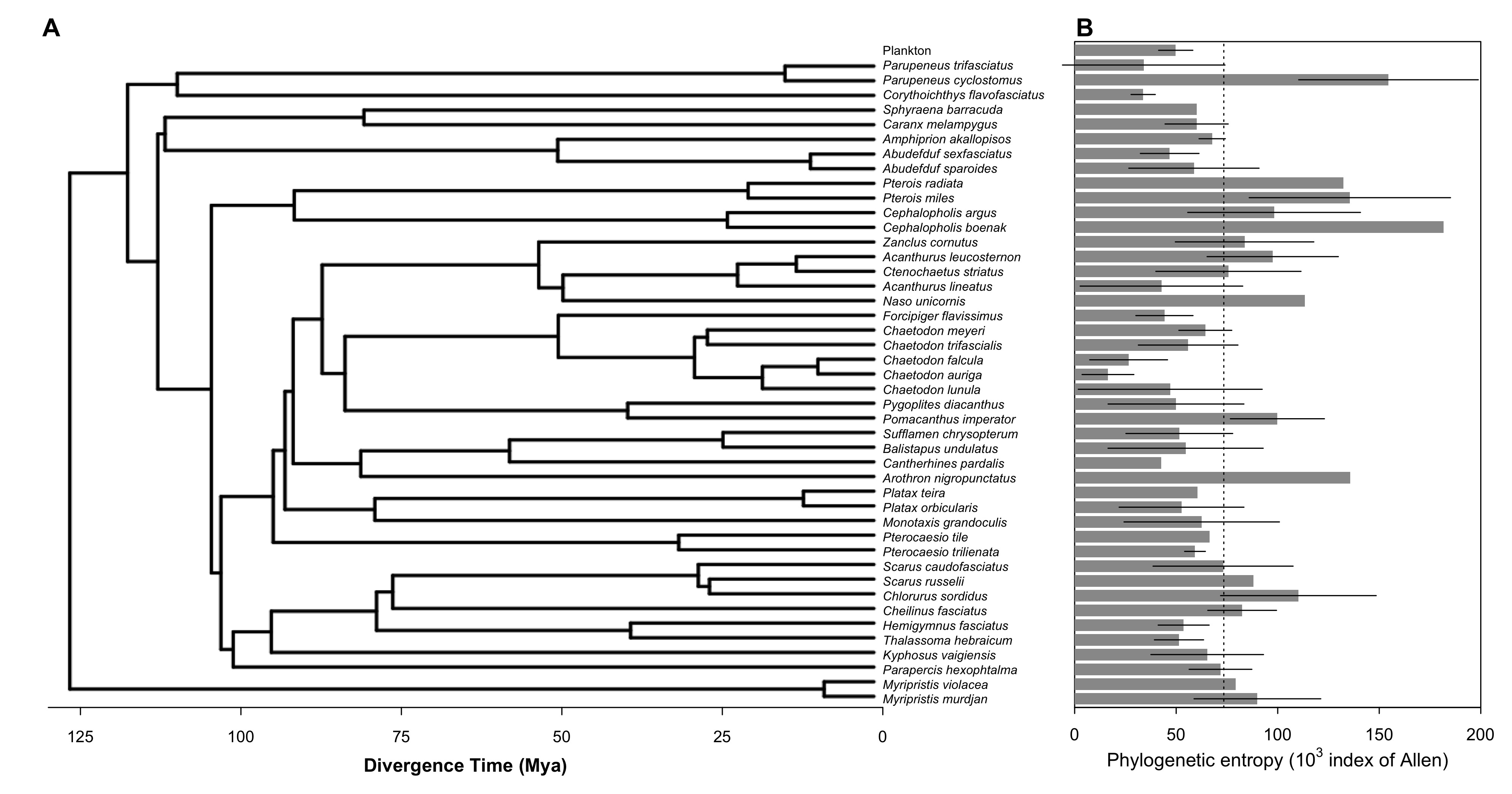


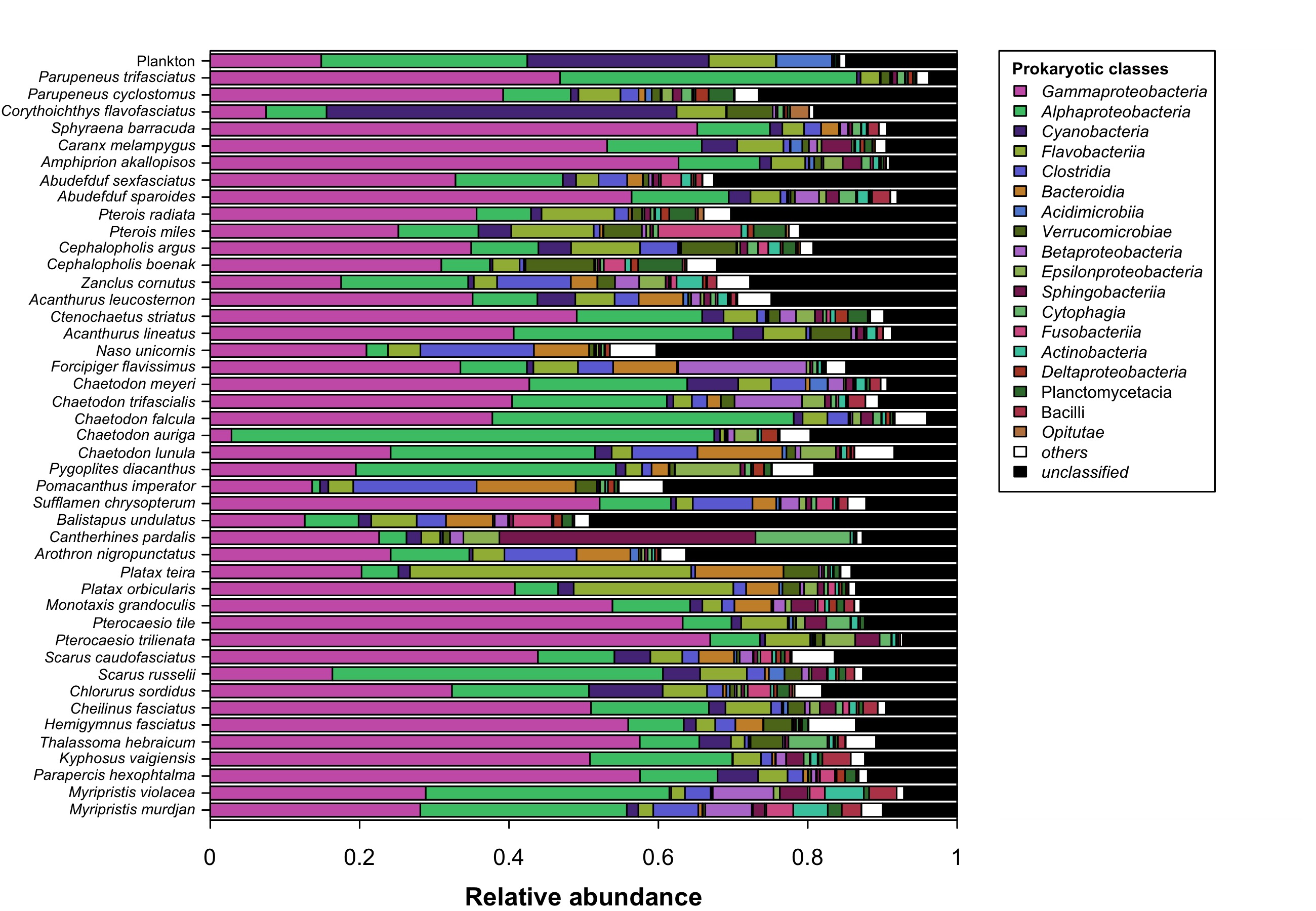


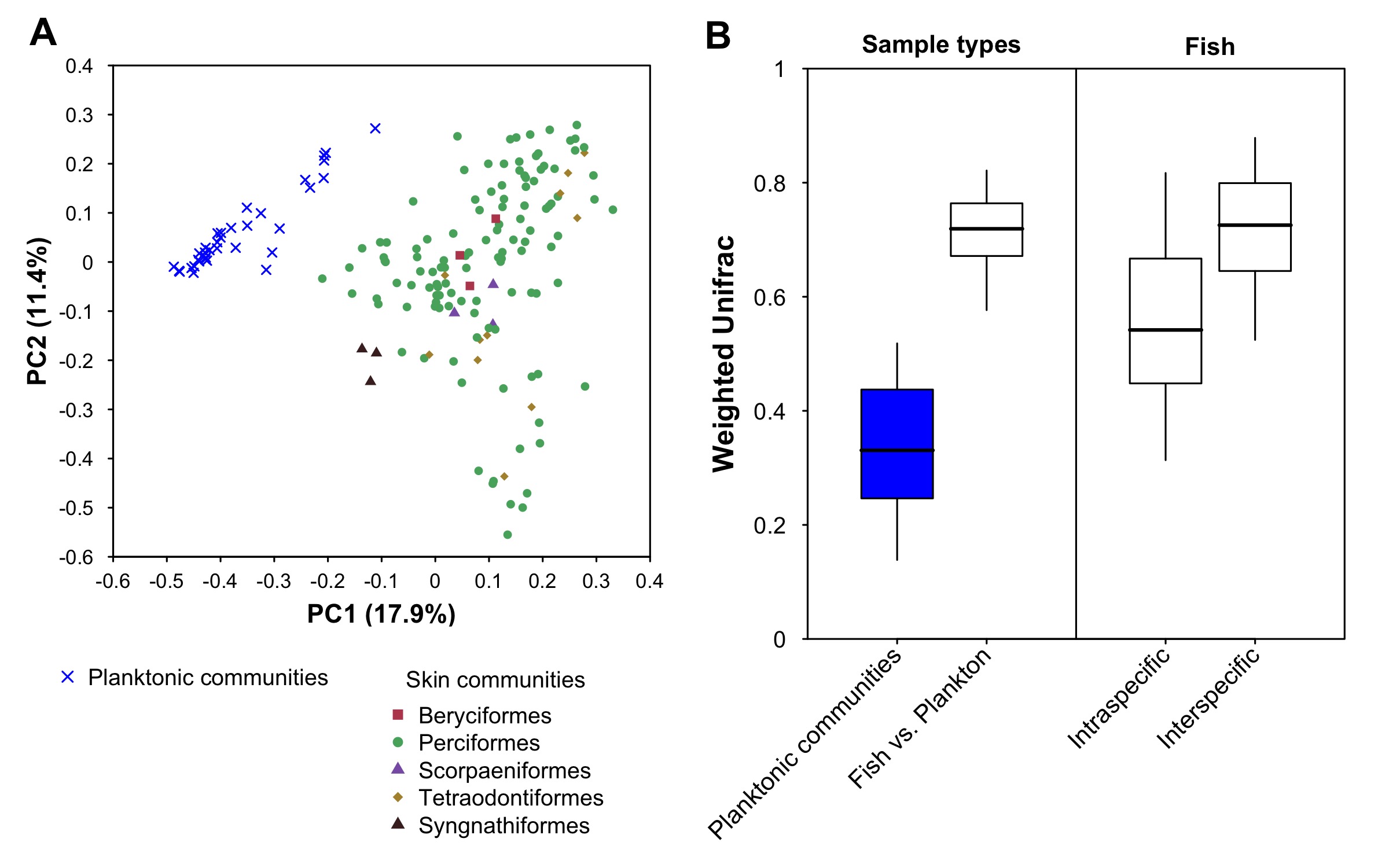


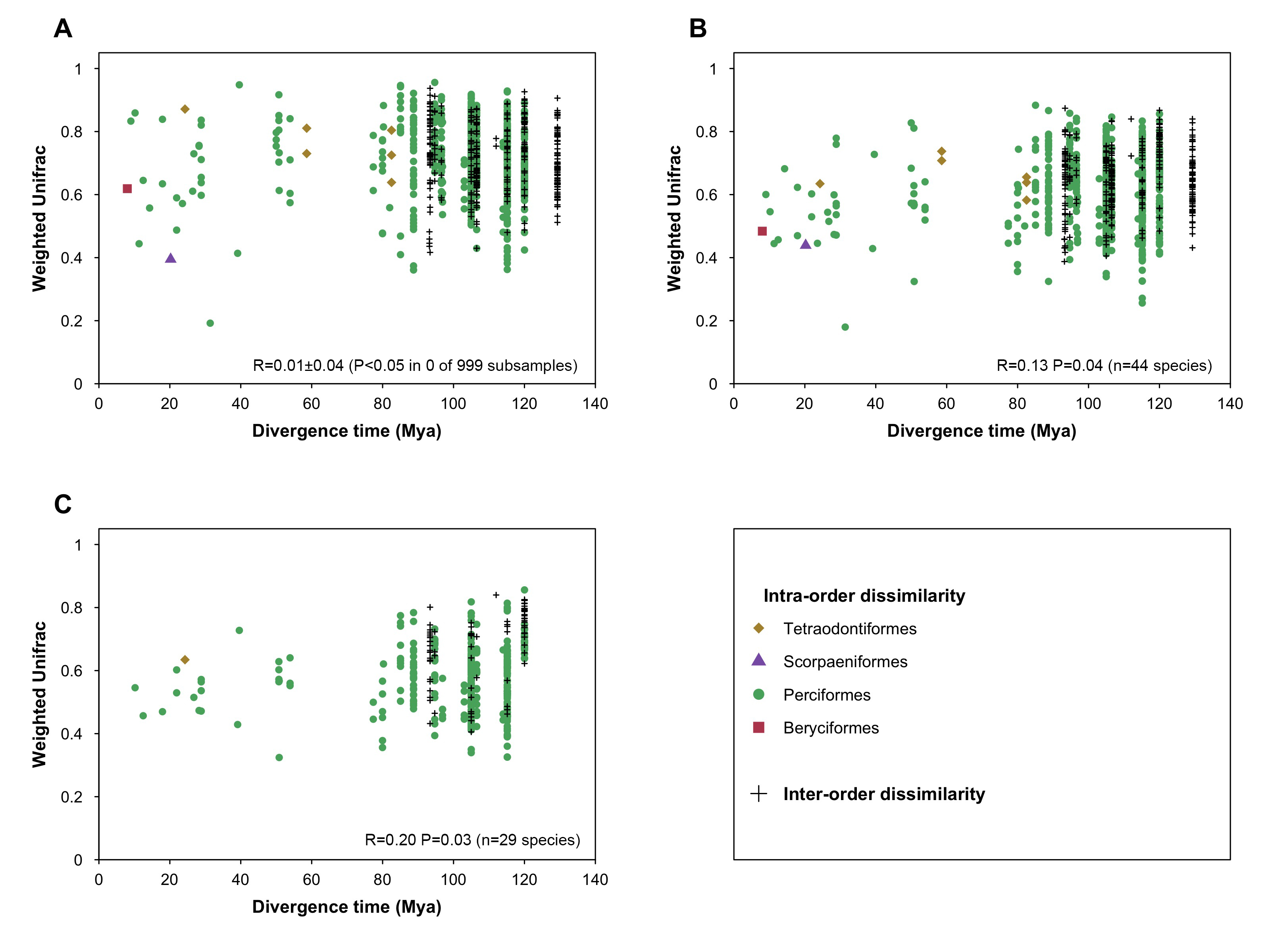

Supplement: Supplementary file 1 — Supplementary information S1 to S13. (DOCX 8832 kb) [file 40168_2018_530_MOESM1_ESM.docx]
